# *Magnolia officinalis* (L.) Bark Extract Counteracts Oxidative Brain Injury: A Proteomic Investigation into Neuroprotective Mechanisms

**DOI:** 10.3390/ijms27083350

**Published:** 2026-04-08

**Authors:** Laura Beatrice Mattioli, Roberto Stella, Caterina Peggion, Stefano Cagnin, Alice Pifferi, Elisabetta Miraldi, Giorgio Cappellucci, Giulia Baini, Luca Camarda, Roberta Budriesi, Maria Frosini

**Affiliations:** 1 Dipartimento di Farmacia e Biotecnologie, Alma Mater Studiorum Università di Bologna, Via Belmeloro 6, 40126 Bologna, Italy; laurabeatrice.mattioli@unibo.it (L.B.M.); l.camarda@unibo.it (L.C.); roberta.budriesi@unibo.it (R.B.); 2 Centro Interdipartimentale di Ricerca Industriale—CIRI Scienze Della Vita e Tecnologie per la Salute, Alma Mater Studiorum Università di Bologna, Via Belmeloro 6, 40126 Bologna, Italy; 3 Dipartimento di Chimica, Istituto Zooprofilattico Sperimentale Delle Venezie, Viale dell’Università 10, 35020 Legnaro, Italy; rstella@izsvenezie.it; 4 Dipartimento di Biologia, Università di Padova, via Ugo Bassi 58/b, 35131 Padova, Italy; caterina.peggion@unipd.it (C.P.); stefano.cagnin@unipd.it (S.C.); 5 CIR-Myo Myology Center, Università di Padova, via Ugo Bassi 58/b, 35131 Padova, Italy; 6 Dipartimento di Scienze Della Vita, Università di Siena, Via Aldo Moro 2, 53100 Siena, Italy; alice.pifferi2@unisi.it; 7 Dipartimento di Scienze Fisiche, Della Terra e dell’Ambiente, Università di Siena, Via Laterina 8, 53100 Siena, Italy; elisabetta.miraldi@unisi.it (E.M.); giorgi.cappellucci@student.unisi.it (G.C.); giulia.baini2@unisi.it (G.B.)

**Keywords:** *Magnolia officinalis* (L.), oxidative stress, proteomics, neuroprotection, age-related disorders, phytochemicals

## Abstract

Neurodegenerative diseases involve progressive neuronal loss associated with oxidative stress (OS) and inflammation. Given the limited efficacy of current therapies, natural compounds with multitarget neuroprotective potential are of growing interest. In this study, we investigated the neuroprotective effects of a standardized *Magnolia officinalis* (L.) bark extract (MOE) in rat brain cortical slices exposed to hydrogen peroxide-induced OS. MOE significantly recovered tissue viability and reduced ROS and malondialdehyde levels caused by OS while attenuating caspase-3, -8, and -9 activation, suggesting modulation of intrinsic and extrinsic apoptotic pathways. Shotgun proteomics using LC-HRMS/MS identified OS-induced protein expression changes reversed by MOE, with fourteen of thirty-three altered proteins rescued by MOE co-treatment. These proteins participate in several processes, including neuronal survival, OS response, and proteostasis. Bioinformatic analysis demonstrated that genes responsible for protein synthesis regulated by MOE are subjected to transcriptional regulation by factors associated with OS, including FOXO4, NRF2, and SP1. The present findings support the hypothesis that MOE exerts multitarget neuroprotective effects by modulating key proteins involved in OS responses and neuronal survival in an acute ex vivo oxidative injury model, suggesting potential relevance for mechanisms associated with NDs.

## 1. Introduction

Neurodegenerative diseases (NDs) constitute a pressing global public health issue, encompassing a diverse spectrum of progressive disorders characterized by the progressive degeneration and loss of neuronal structure and function within the central (CNS) and/or peripheral nervous system. These pathological processes result in a progressive deterioration of cognitive abilities, memory, behavior, sensory perception, and motor coordination, substantially impairing patient quality of life and imposing considerable socioeconomic burdens [1]. Representative examples include chronic NDs, such as Alzheimer’s disease (AD), Parkinson’s disease (PD), Huntington’s disease (HD), and amyotrophic lateral sclerosis (ALS), as well as acute neurological conditions, such as cerebral ischemia, traumatic brain injury, and spinal cord injury. Each of these disorders exhibits distinct clinical manifestations and is underpinned by unique, yet often overlapping, pathogenic mechanisms.

NDs share several defining pathological characteristics, including the accumulation of aberrant proteins that form intra- or extracellular inclusions, such as amyloid-beta plaques and tau tangles in AD, alpha-synuclein aggregates in PD, and mutant huntingtin protein in HD. These conditions are also marked by impaired proteostasis, cytoskeletal abnormalities, DNA and RNA damage, oxidative stress (OS), mitochondrial dysfunction, and chronic inflammation. Together, these mechanisms disrupt synaptic and neuronal network function, ultimately leading to progressive neuronal cell death, resulting in the characteristic symptoms associated with each previously specified disease [2]. Aging stands as the predominant risk factor, although genetic predisposition, environmental toxins, lifestyle factors like diet and physical activity, and certain medical conditions also contribute to ND susceptibility [3]. Addressing NDs poses a substantial modern medical challenge, particularly considering the aging population and the prevalence of multiple concurrent health conditions among the elderly.

The therapeutic management of NDs continues to present significant challenges, with limited progress in the development of effective disease-modifying therapies [4]. Conventional pharmacological treatments typically focus on single molecular targets, which do not adequately address the complex and multifactorial nature of these disorders, primarily due to several inherent challenges. These include the restrictive nature of the blood–brain barrier, which restricts drug delivery to the central nervous system, and the frequent occurrence of adverse side effects associated with existing treatments. These factors contribute to suboptimal clinical outcomes and reduced patient survival rates [5].

In this context, there is an increasing scientific interest in natural products from plants due to their broad spectrum of pharmacological activities and potential for multitarget interactions. Bioactive molecules derived from natural sources such as plants, herbs, and fruits constitute valuable pharmacological sources, and numerous studies have underscored their neuroprotective properties [6,7].

Among the natural compounds of therapeutic interest, *Magnolia officinalis* (L.) bark extract (MOE) has gained attention for its neuroprotective potential, primarily attributed to its key bioactive constituents, magnolol and honokiol [8]. These compounds exhibit multiple beneficial pharmacological activities, including potent antioxidant, anti-inflammatory, and neurogenic effects [8,9,10,11], along with the ability to inhibit amyloidogenic peptide aggregation and promote the disassembly of preformed aggregates [12], thereby supporting their potential as therapeutic agents against neurodegenerative processes.

Shotgun proteomic analysis based on label-free quantification by liquid chromatography coupled with high-resolution tandem mass spectrometry (LC-HRMS/MS) is a powerful approach that enables comprehensive, unbiased profiling of protein expression in complex biological samples, capable of identifying and quantifying thousands of proteins in a single run, thereby revealing treatment-induced changes linked to OS, inflammation, neuronal survival, and synaptic plasticity [13,14,15,16,17,18]. In the present study, the neuroprotective effects of MOE, recently characterized by a high content of magnolol and honokiol, against OS-induced damage in rat brain slices were evaluated. To deeply investigate the underlying molecular mechanisms of neuroprotection, a shotgun proteomic analysis was employed to assess protein expression changes induced by OS, with particular attention to those normalized by MOE treatment. Additionally, a bioinformatic approach was employed to identify transcription factors that may be responsible for gene expression regulation, thereby modulating protein expression alterations associated with MOE. It was shown that MOE treatment has a protective effect on OS-induced injury, also modulating both intrinsic and extrinsic apoptotic pathways. Results support the idea that a proteomic approach might be a valuable platform for discovering promising candidates for further research in NDs.

## 2. Results

### 2.1. Effects of MOE Per Se on Rat Brain Slices Viability

**Figure 1:**
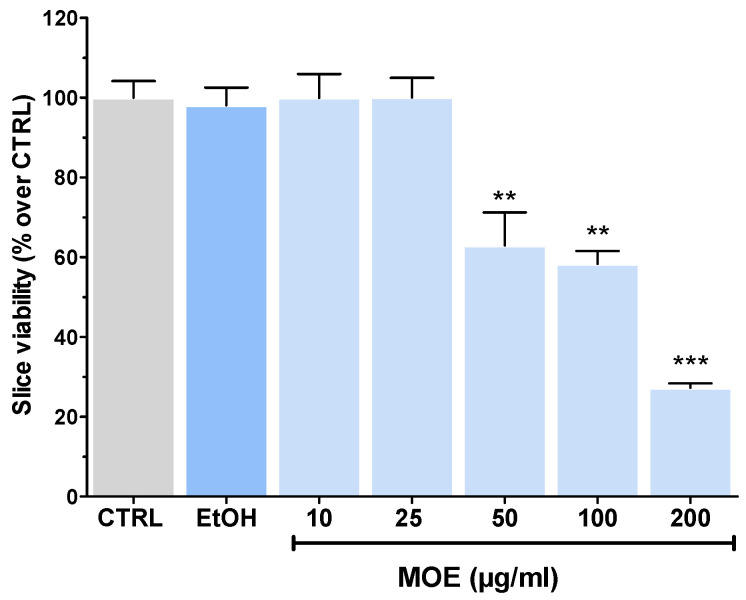
Effect of MOE treatment (10–200 µg/mL) on the viability of rat brain slices after 2 h of treatment. Data are shown as mean ± SEM (*n* = 3–4). CTRL, controls; EtOH, tissue-treated ACSF containing the highest percent value of ethanol (2%), as in MOE 200 μg/mL. Statistical analysis was performed with ANOVA, followed by a Bonferroni post hoc test. ** *p* < 0.01, *** *p* < 0.001 vs. CTRL.

The effects of MOE per se were initially tested to determine whether it could affect the viability of rat brain slices. Treatment with 2% EtOH (the highest concentration in MOE samples at 200 µg/mL) did not impair tissue viability (Figure 1 and Supplementary Data Figure S1), similarly to the treatments with 10 µg/mL and 25 µg/mL MOE. In contrast, starting from 50 µg/mL MOE, a significant decrease in viability was detected, with a maximum reduction of 72.7% observed at 200 µg/mL MOE (Figure 1). Considering these results, the neuroprotective activity of MOE was evaluated at concentrations below 25 µg/mL.

### 2.2. MOE-Mediated Neuroprotection Under OS Conditions

**Figure 2:**
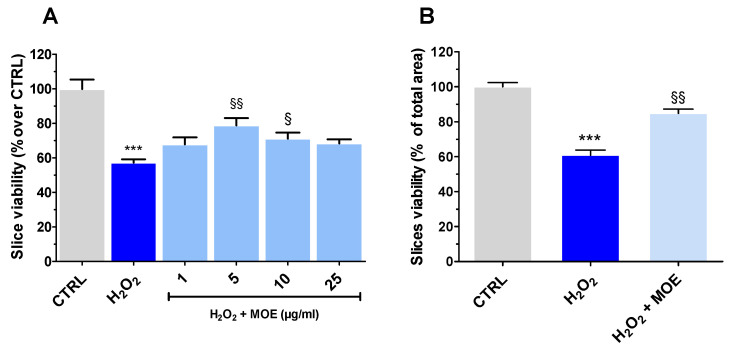
Effect of MOE on H_2_O_2_ (20 mM, 1 h)-induced injury in rat brain slices. (**A**): Viability assessed by using an MTT assay. (**B**): Analysis of healthy versus damaged areas using ImageJ in the presence of MOE at 5 µg/mL, the most active concentration in the MTT assay. Data were reported as mean ± SEM (*n* = 3–4). Statistical analysis was performed by ANOVA, followed by a Bonferroni post hoc test. *** *p* < 0.001 vs. CTRL, § *p* < 0.05, §§ *p* < 0.01 vs. H_2_O_2_.

OS (H_2_O_2_, 20 mM, 1 h) caused a significant reduction in slices’ viability (−42.70 ± 1.90% MTT assay, −39.10 ± 2.95% analysis with ImageJ version 1.54g) (Figure 2A and Figure 2B, respectively). However, pretreatment of slices with MOE (1–25 μg/mL) exerted neuroprotective effects. A hormetic-like recovery in tissue viability was obtained following MOE treatment with the maximal effect at 5 µg/mL (Figure 2A,B). Treatment with 10 µg/mL of MOE was still protective, whereas both the lower (1 µg/mL) and higher concentrations (25 µg/mL) were ineffective against OS.

To further investigate the neuroprotective effects of MOE, the duration of H_2_O_2_-induced injury was extended to 2 h. Despite the prolonged OS, MOE treatment tended to improve cell viability compared to H_2_O_2_ alone, with the strongest effect observed at 5 µg/mL; however, none of these differences reached statistical significance (Supplementary Figure S2). These findings suggest that the extent of damage caused by a two-hour oxidative insult may be too severe to permit substantial recovery under the applied MOE treatment conditions.

### 2.3. MOE Reverted ROS Formation and Lipid Peroxidation Caused by OS

As OS leads to the accumulation of ROS in the cytosol, their levels were evaluated in tissues exposed to H_2_O_2_, along with the ability of MOE to counteract their formation. MOE was used at concentrations of 1 (ineffective), 5, and 10 µg/mL (neuroprotective).

**Figure 3:**
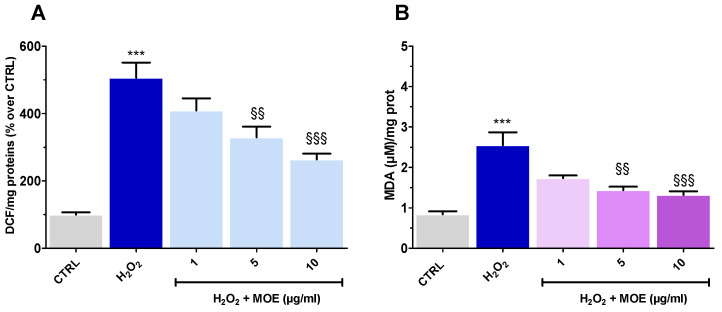
Effect of MOE (1–10 µg/mL) on H_2_O_2_ (20 mM for 1 h)-induced ROS (**A**) and MDA (**B**) formation in rat brain slices. Data were reported as mean ± SEM (*n* = 3–4). Statistical analysis was performed by ANOVA, followed by a Bonferroni post hoc test. *** *p* < 0.001 vs. CTRL, §§ *p* < 0.01, §§§ *p* < 0.001 vs. H_2_O_2_.

OS caused a striking increase in ROS formation, which was, however, progressively reverted by increasing MOE concentrations (Figure 3A).

This reduction exhibited statistical significance at concentrations of 5 and 10 µg/mL of MOE, while 1 µg/mL proved ineffective. Interestingly, the antioxidant capacity of MOE, i.e., its ability to neutralize free radicals, assessed by a DPPH assay, showed an IC_50_ of 326.1 µg/mL, a value much higher than that of ascorbic acid (0.02 µg/mL), used as a reference compound (Supplementary Data Figure S3). These findings suggest that the reduction in ROS levels observed at low MOE concentrations is unlikely to be solely due to a direct radical-scavenging effect. Rather, it is more likely mediated by the activation of endogenous antioxidant pathways or other indirect cytoprotective mechanisms. OS also leads to the formation of MDA, a byproduct of lipid peroxidation. This process occurs when ROS attack polyunsaturated fatty acids in cell membranes, resulting in the generation of malonaldehyde as a stable end-product [19]. As shown in Figure 3B, H_2_O_2_ induced a marked increase in MDA levels, reflecting significant lipid peroxidation. Treatment with MOE at concentrations of 5 and 10 µg/mL reduced these levels, suggesting a potential protective effect. Although the lowest MOE concentration (1 µg/mL) showed a similar trend, this effect was not statistically significant.

### 2.4. MOE-Induced Changes in Caspase Activity

Several pathways of apoptosis are involved in the induction of cell death, including the mitochondria-mediated and the extrinsic receptor-mediated pathways. Caspase-9 and -8 play an essential role in these pathways with the ability to activate caspase-3 and initiate DNA fragmentation [20,21,22]. To investigate the involvement of these caspases in neuroprotection mediated by MOE, we evaluated their activity in tissue lysates of MOE-treated rat brain slices. Only the most active concentration of MOE (5 µg/mL) was tested, as this consistently showed the strongest neuroprotective effect across multiple assays.

**Figure 4:**
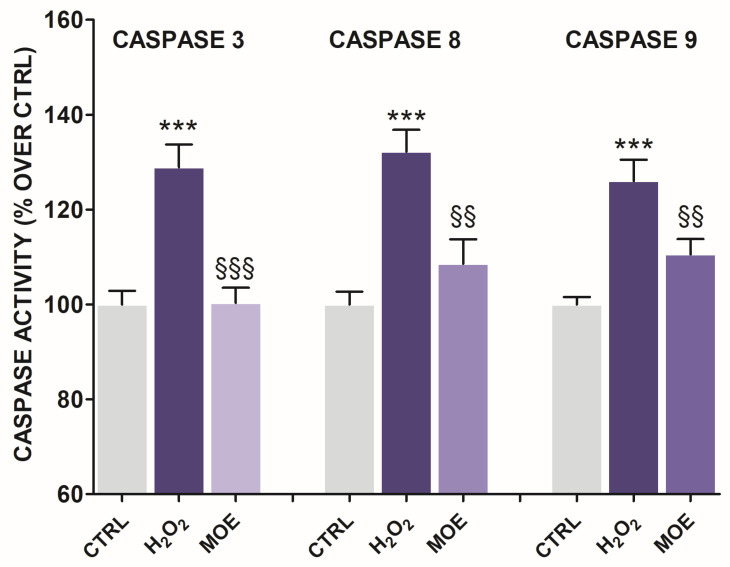
Effects of MOE (5 µg/mL) on H_2_O_2_ (20 mM for 1 h)-induced caspase-3, -8, and -9 increased activity in rat brain slices. Data are reported as mean ± S.E.M. (*n* = 3). Statistical analysis was performed by ANOVA, followed by a Bonferroni post hoc test. *** *p* < 0.001 vs. controls, §§§ *p* < 0.001, §§ *p* < 0.01 vs. H_2_O_2_.

The exposure to H_2_O_2_ resulted in a significant activation of all three caspases compared to untreated brain slices, consistent with the involvement of both intrinsic and extrinsic apoptotic pathways (Figure 4). Co-treatment with MOE effectively suppressed caspase activation, resulting in caspase-3 activity levels comparable to those observed in the control group (Figure 4), thus supporting the neuroprotective potential of MOE in mitigating OS-induced apoptosis.

### 2.5. Brain Slices Proteomic Analysis

**Figure 5:**
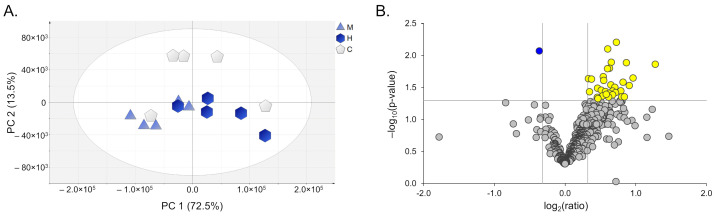
PCA score plot comparing the proteomic profile of the three groups under investigation (**A**). Pentagon, controls; hexagon, H_2_O_2_; triangle, H_2_O_2_ + MOE (5 µg/ml). (**B**) Volcano plot correlating the statistical significance to the fold change. Down-regulated proteins are reported in blue, up-regulated proteins are reported in yellow and proteins whose amount was not significantly modulated following H_2_O_2_ treatment are depicted in grey, according to the *p*-value and fold-change cut-offs considered.

To elucidate the alterations in brain slices treated with H_2_O_2_ compared to their untreated counterpart and the mechanism by which MOE exerts its neuroprotective effect (5 µg/mL), we conducted a proteomic analysis using five different rats for each condition (five controls, five H_2_O_2_-treated, and five H_2_O_2_/MOE-treated). We identified 869 proteins with at least one peptide and a false discovery rate below 1%, and 746 of these proteins were identified with two peptides (Supplementary Table S1). Of these, 536 were properly quantified with at least one unique peptide and a CV% of less than 25% among the QV samples prepared and analyzed during the analytical session. Relative quantification data coming from the non-targeted proteomics analysis were subjected to PCA (Figure 5A). PCA shows that the brain slices of the three groups under comparison are not well separated in distinct clusters; however, a trend is observable. Indeed, the control and H_2_O_2_/MOE-treated samples are slightly separated from the H_2_O_2_-treated samples, along the first principal component (PC1, x-axis), indicating that the overall proteomic profile is similar, but a specific difference can be present in the dataset.

To highlight which proteins are differentially expressed among the considered conditions, we applied an ANOVA analysis considering the 536 quantified proteins among the three sample groups. A total of 33 proteins were significantly differentially expressed (*p*-value < 0.05) in the brain treated with H_2_O_2_ (H_2_O_2_/control ratio >1.25; 32 proteins and H_2_O_2_/control ratio < 0.8; 1 protein) (Table 1).

These results are reported using a volcano plot, in which the fold change and the statistical significance are combined (Figure 5B).

To elucidate the potential mechanism of action of the MOE, our focus was directed toward proteins whose expression was exclusively induced by H_2_O_2_ treatment, while their levels did not change upon inclusion of MOE in the treatment. Interestingly, 72% of the differentially expressed proteins (18 out of 33 proteins) responded to the MOE treatment (Table 2).

**Figure 6:**
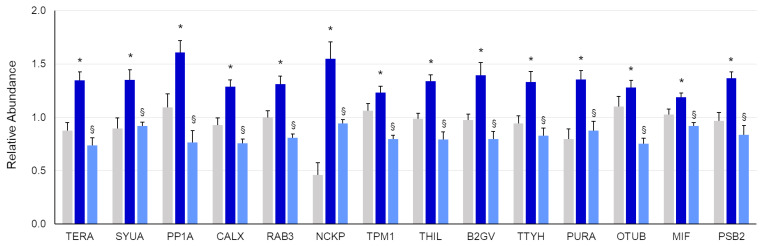
Bar diagram reporting relative quantification values (mean ± SEM) of proteins analyzed by targeted PRM validation. Gray bars: controls; blue bars: H_2_O_2_; light blue bars: H_2_O_2_ + MOE (5 µg/mL). Among the 18 proteins whose amount was affected by H_2_O_2_ treatment, 14 were found to be unaltered following the addition of MOE (* H_2_O_2_ vs. control, *p* < 0.05; § H_2_O_2_/MOE vs. H_2_O_2_), *p* < 0.05, one-way ANOVA).

**Figure 7:**
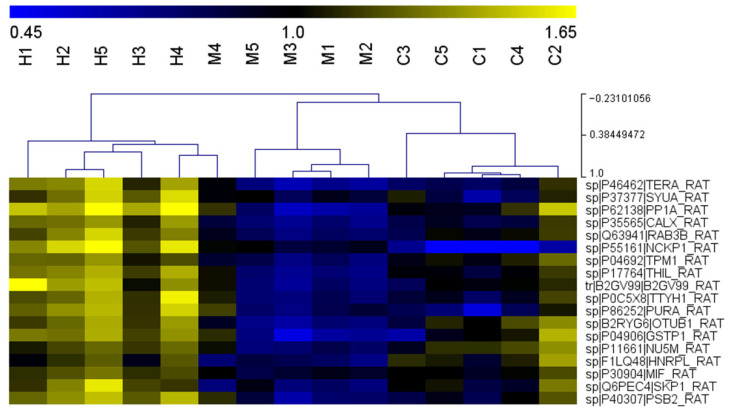
Hierarchical clustering on quantitative data coming from targeted proteomic analysis. In the heat map, 18 protein targets whose amount was affected by H_2_O_2_ treatment and found to be unaltered by MOE (5 µg/mL) presence are reported. Rows: UniProtKB protein accession number; columns: experimental groups; color key indicates relative protein amount, yellow: up-trend; blue: down-trend. Protein amount was average-normalized prior to analysis. C, controls; H, H_2_O_2_; M, H_2_O_2_ + MOE.

These proteins were chosen for a subsequent targeted validation study by an MS-based parallel reaction monitoring (PRM) approach. With this analysis, 14 proteins were confirmed to be significantly altered by the H_2_O_2_ treatment and protected by the presence of MOE (Figure 6). Cluster analysis using relative quantification data derived from targeted PRM analysis shows a clear separation of the H_2_O_2_-treated samples from the control- and MOE-treated ones (Figure 7).

**Figure 8:**
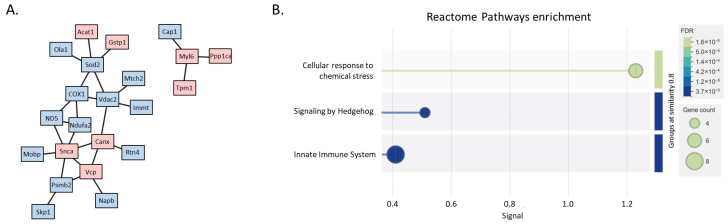
Protein–protein interaction network and functional enrichment of 33 altered proteins. (**A**) Protein–protein interaction (PPI) network generated by STRING using default parameters. Nodes represent proteins, with those reconfirmed by PRM highlighted in pink. Two main clusters are visible, indicating distinct interaction modules, while some proteins remain as isolated nodes. (**B**) Functional enrichment analysis of the protein set showing significantly enriched biological processes. The dot plot displays gene counts (circle size) and false discovery rate (FDR, color gradient), highlighting pathways related to cellular response to chemical stress and other key processes.

All 33 proteins were uploaded into STRING to assess their functional associations. The analysis revealed two main clusters (highlighted in pink for proteins reconfirmed by PRM), indicating distinct interaction networks (Figure 8). Not all proteins were part of the clusters; some remained as single entities. Nevertheless, the PPI enrichment *p*-value of 2.61 × 10−^6^ indicates a higher-than-random level of connectivity among these proteins. Further enrichment analysis showed that these proteins are involved in the cellular response to chemical stress.

### 2.6. Factors Regulating the Expression of Proteins Responding to H_2_O_2_ and MOE Treatment

We further explored whether alterations in protein expression following OS may be associated with transcriptional gene regulation mediated by specific transcription factors (TFs). A computational analysis was performed to verify whether common TFs regulate the expression of genes coding for the altered proteins. Analyzing each promoter sequence, we evidenced that each promoter could bind to several TFs (Supplementary Table S2).

**Figure 9:**
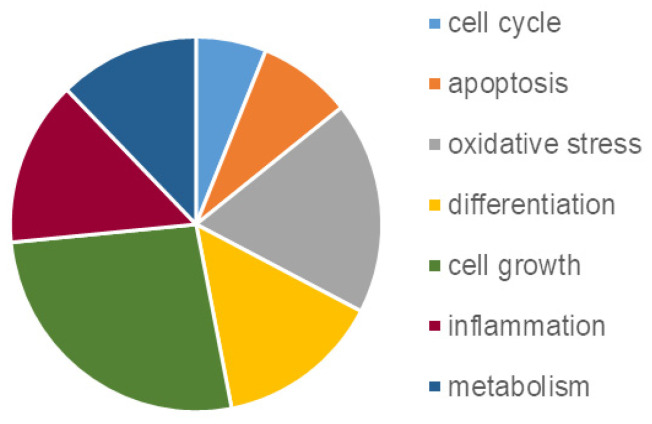
Pie chart representing the gene ontology functional categorization of enriched TFs reported in Table S3, drawn by using the EnrichR webtool (see Section 4).

Considering the most enriched TFBS, it is interesting that several of them are associated with brain function and development, such as Zic family member 2 (ZIC2), cAMP Response Element-Binding Protein (CREB), hepatocyte nuclear factor 3-beta (HNF3B), also known as Forkhead box protein A2 (FOXA2), E74-like factor 1 (ELF1), and Forkhead box (FOX) (Supplementary Table S3). The pie chart in Figure 9 depicts the major functions in which such TFs are involved. Interestingly, several of them contribute to the OS response, such as Forkhead Box O4 (FOXO4), Nuclear factor erythroid 2-related factor 2 (NRF2), Specificity Protein 1 (SP1), and Nuclear Transcription Factor Y (NFY) [23,24] (Supplementary Table S4).

## 3. Discussion

The present study aimed to assess the neuroprotective properties of a *Magnolia officinalis* (L.) bark extract (MOE), recently characterized for its high magnolol and honokiol content, toward OS-mediated injury in rat brain slices. This experimental acute ex vivo model preserves native cytoarchitecture and local synaptic activity and reproduces key pharmacological and genetic responses observed in vivo in a normal or injured brain tissue context without complication from brain penetration or metabolic stability, thus being widely used to investigate neuropathological mechanisms and screen therapeutic compounds [25,26,27]. To reproduce OS-mediated injury, a challenge with hydrogen peroxide was performed (H_2_O_2_ 20 mM for 1 h, i.e., an injury causing ≈ 50% damage), as it provides a reproducible and intermediate level of toxicity that is sufficient to detect protective effects while avoiding excessively severe damage that would be largely irreversible or, conversely, too mild to reveal meaningful protection. This paradigm, however, reflects early oxidative stress-mediated neuronal dysfunction rather than the extensive cell loss characteristic of late-stage neurodegeneration, and by monitoring markers such as mitochondrial activity and enzymatic function shortly after H_2_O_2_ treatment, early events in the degenerative cascade are captured. This widely used “acute” experimental model may not be relevant to the protracted degeneration observed in patients characterized by low-grade chronic inflammation and OS [28,29]. Nevertheless, it has greatly increased our knowledge of the biochemical mechanisms of neuronal cell death in NDs and is still very helpful to make a preliminary screening of potentially neuroprotective drugs as well as to characterize their mechanism of action. To highlight MOE use as a possible neuroprotective drug, its administration after inducing OS would probably better resemble the in vivo condition. In this regard, it should be considered that this approach is particularly useful in acute neurodegenerative settings, such as ischemic stroke or traumatic brain injury, where rescuing damaged tissue after the insult is critical, and natural compounds may be effective when administered post-injury [30]. In this study, we performed the pretreatment approach because it more accurately reflects the therapeutic goal of intervening during the early, asymptomatic stages of neurodegeneration, when preventive strategies are most likely to be effective. Results showed that MOE exerted neuroprotection at concentrations ranging from 5 to 10 µg/mL, while this effect was less pronounced under a 2-h H_2_O_2_ challenge, which helps define the conditions under which neuroprotection may occur. The neuroprotective MOE concentrations correspond to 0.5–1 µM honokiol and 0.3–0.6 µM magnolol based on the quantitative analysis [11]. These results are consistent with data from cellular models of NDs, where honokiol and magnolol exhibit neuroprotective effects at concentrations typically ranging from 0.5 to 5 µM and 2.5 to 10 µM, respectively, mitigating OS, inflammation, and apoptosis induced by neurotoxins such as β-amyloid and glutamate [31,32]. In vitro neuroprotection is also supported by in vivo studies demonstrating that administration of these compounds in animal models of neurodegeneration reduces cognitive deficits, neuroinflammation, and neuronal loss at brain concentrations in the low micromolar range [33]. Among the mechanisms underlying these effects, which are still under debate, the potentiation of the GABAergic [34,35] and endocannabinoid systems [36,37,38] has been proposed. Although the present study did not directly assess the involvement of GABA or cannabinoid receptors in MOE-mediated neuroprotection, their contribution to the observed effects cannot be excluded and warrants further investigation. Altogether, the present findings underline the neuroprotective potential of honokiol and magnolol in an ex vivo model, and their multimodal actions, encompassing antioxidant, anti-inflammatory, GABAergic, and cannabinoid receptor-mediated mechanisms, support their development as promising candidates for modulating pathways associated with oxidative and inflammatory damage relevant to neurodegenerative conditions. However, the present data showed that MOE caused a reduction in brain slice viability at concentrations higher than 25 µg/mL, a value two to four times higher than the range shown to be neuroprotective. This observation agrees with data from the literature reporting that honokiol has neuroprotective effects at low doses while causing cytotoxicity at higher concentrations [37,39], matching the behavior of many GABA_A_ [40] as well as CB1 and CB2 agonists [41]. Furthermore, the possibility that the non-linear, hormetic-like response may reflect the combined action of multiple bioactive compounds within the extract, each with distinct potency and targets, as well as biological threshold effects, cannot be ruled out. At moderate concentrations, these compounds may collectively activate protective pathways, whereas at higher doses, some components may overwhelm cellular homeostasis or engage cytotoxic mechanisms, a possibility that warrants further investigation.

The formation of ROS is a major contributor to neuronal damage and plays a key role in the development of several neurological conditions, including NDs as well as mood disorders (e.g., anxiety, stress, and depression) [42,43]. In the first part of this study, exposure of rat brain slices to hydrogen peroxide resulted in a marked loss of viability, likely due to excessive ROS generation. ROS production disrupts endogenous antioxidant defenses and induces oxidative damage to membrane lipids, proteins, and DNA. Furthermore, it plays a pivotal role in regulating apoptotic pathways mediated by mitochondria, death receptors, and the endoplasmic reticulum [44,45]. Consistent with these mechanisms, OS in our model led to a significant increase in ROS and malondialdehyde (MDA) levels, along with elevated caspase-3, -8, and -9 activity, indicating the activation of both intrinsic and extrinsic apoptotic pathways. Notably, treatment with MOE at 5 and 10 µg/mL effectively prevented these effects, and neuroprotective activity was observed at concentrations approximately 60-fold lower than those required to elicit antioxidant effects. This suggests that mechanisms other than direct ROS scavenging may contribute to the observed neuroprotection, with the activation of endogenous antioxidant pathways being a plausible, though not directly confirmed, mechanism.

OS is known to cause protein modifications, such as oxidation, misfolding, aggregation, and alterations in expression levels through the activation of specific transcription factors. Such changes in protein levels could be useful indicators of pathologies, including NDs. In this regard, pharmacological strategies aimed at restoring the normal expression levels of such proteins could represent a potential approach to address diseases related to oxidative damage. To date, no studies have investigated the proteomic changes in brain slices exposed to hydrogen peroxide and the neuroprotective effects of MOE, highlighting the critical need to explore these molecular alterations to better understand OS-induced damage and the mechanisms underlying MOE neuroprotection. Specifically, in this study, we found a group of 14 proteins involved in different cellular functions (such as cell signaling, actin cytoskeleton dynamics, cellular stress, and immune response) that showed OS-related altered expression levels. This suggests that such proteins may be susceptible to oxidative damage or contribute to the cellular response to ROS in this ex vivo model. Additionally, we discovered that the presence of MOE together with OS counteracted the alteration of the amount of such proteins, indicating that it may have a protective function by boosting cellular repair mechanisms, stimulating intrinsic cellular antioxidant defenses, stabilizing protein function, and preserving cellular homeostasis in the face of OS.

Some of these proteins play a crucial role in the nervous system, and their unbalanced expression is related to various pathologies. Among them, we identified α-synuclein, a key player in PD and, in general, in synucleinopathies. Its expression is known to increase under stress conditions such as OS, ER stress, and inflammation, as part of a cellular stress response mechanism [46]. However, chronic stress can lead to α-synuclein pathological accumulation, contributing to disease progression through transcriptional, epigenetic, or post-translational mechanisms [47]. In our study, MOE treatment appeared to modulate α-synuclein levels, suggesting its potential to mitigate, at least in vitro, α-synuclein accumulation and providing a promising strategy for preventing or attenuating synucleinopathy-related neurodegeneration [48]. Another protein whose dysfunction has been linked to several human diseases, including NDs (ALS, HD, Charcot–Marie–Tooth disease) and cancers [49,50,51,52,53,54], is the transitional endoplasmic reticulum ATPase, also known as valosin-containing protein (VCP). VCP is a key regulator of mitochondrial quality control [55], evolutionarily conserved and predominantly cytoplasmic AAA+ ATPase involved in multiple cellular processes to ensure proteostasis through either the ubiquitin-proteasome system or the autophagy/lysosomal route and endoplasmic reticulum-associated degradation [56,57]). VCP expression and activity were shown to be tightly regulated during stress to ensure proper protein quality control and to prevent cell damage or death. As VCP, calnexin serves as a chaperone, helping in protein folding, quality control in the endoplasmic reticulum, and stress responses [58]. In addition to its chaperone function, calnexin has been implicated in calcium homeostasis [59], phagocytosis [60], and ER-associated degradation [61]. Mounting evidence suggests that calnexin itself acts as a sensor to regulate apoptosis induced by ER stress [62,63,64]. The increased expression of calnexin consequently to OS was already reported in an in vitro model of human diploid fibroblasts obtained from young individuals, together with an age-related decline in OS-induced calnexin expression, thus suggesting that calnexin is closely related to the aging process, and it might contribute to establishing a cytoprotective state in a variety of human age-related diseases [65].

Our data suggest that OS-affected mitochondrial metabolism could be rescued by MOE, as indicated by changes in the mitochondrial acetyl-CoA acetyltransferase (ACAT1) protein level, a key enzyme in ketone body metabolism and mitochondrial beta-oxidation. The upregulation of ACAT1 expression was already detected in OLN93 oligodendrocytes after excitotoxicity and OS and attenuated by antioxidant treatment, thus suggesting that its expression is sensitive to cellular redox states and metabolic shifts [66]. For instance, ACAT1 expression may be induced by OS via regulation of nuclear factor kB and hypoxia-inducible factor [66]. Interestingly, ACAT1 activity was found enhanced in diverse human cancer cell lines, thus suggesting ACAT1 as a potential new anti-cancer target [67]. Regulation of ACAT1 by MOE could, therefore, support mitochondrial function and energy metabolism under stress conditions in brain slices.

Another critical protein affected is the proteasome subunit beta type-2 (Psmb2), an essential component of the 20S proteasome core complex, responsible for degrading damaged or misfolded proteins. Psmb2 plays a pivotal role in maintaining proteostasis and regulating immune responses, especially under conditions of OS [68]. Deregulation of Psmb2 has been linked to altered protein clearance in aging and disease, and emerging evidence also implicates it in cancer progression and glioma immunotherapy [69]. Restoration of Psmb2 expression by MOE, seen in the present ex vivo brain preparation, may contribute to improved cellular proteostasis and immune regulation.

Similarly, the observed modulation of S-phase kinase-associated protein 1 (SKP1) by MOE suggests a potential protective effect on protein turnover and neuronal survival. For instance, SKP1 is a core component of the largest class of E3 ubiquitin ligase complex, SCF (Skp1, Cullin 1, a substrate recognizing F-box protein, and Rbx1) that participates in the ubiquitination and degradation of proteins involved in critical pathways such as cell cycle progression and signal transduction. Interestingly, decreased SKP1 expression has been reported in the substantia nigra of PD patients, linking its dysfunction to disease pathogenesis [70].

Pur-alpha is a ubiquitous and evolutionarily conserved DNA- and RNA-binding protein that plays a key role in both transcription and translation and functions as a tumor suppressor [71]. Pur-alpha was found to be essential for CNS development and neuronal survival, and its mutation was linked to neurodevelopmental and neurodegenerative disorders [72]. Although there is currently no evidence in the literature linking its expression to OS, our findings suggest that it may be involved in brain slice cellular stress responses, thus warranting further investigation.

Additional proteins altered by OS and rescued by MOE include the protein phosphatase 1 alpha isoform (PP1A), the small GTPase Rab3B, and the cytokine Macrophage Migration Inhibitory Factor (MIF). PP1A, a serine/threonine phosphatase composed of catalytic and regulatory subunits, is involved in key cellular processes like cell cycle regulation, metabolism, and neuronal functions, including synaptic plasticity, learning, and memory [73]. Our findings revealed an increase in PP1A expression following OS, in contrast with observed data demonstrating that PP1A activation occurs primarily through post-translational modifications rather than changes in expression levels [74]. This discrepancy suggests a possible cell-type or context-dependent regulatory mechanism, especially in rat cortical slices, where OS may trigger a compensatory upregulation in PP1A to counteract aberrant phosphorylation events. Importantly, treatment with MOE rescued PP1A expression, indicating that MOE not only exerts antioxidant effects but may also modulate gene expression or signaling pathways involved in PP1A regulation, thereby supporting neuronal function and protecting against oxidative damage. Overall, these results underscore MOE’s potential as a multitarget neuroprotective agent capable of fine-tuning critical cellular processes disrupted during stress in an ex vivo model of OS.

RAB3B is a small GTPase essential for vesicular trafficking, relying on the binding and hydrolysis of GTP for its proper function [75]. OS can impact the activity of RAB3B by oxidizing critical cysteine residues or interfering with GTP binding and hydrolysis, which can disrupt vesicular trafficking and cellular signaling pathways [76]. However, OS has been shown to regulate the expression of other vesicular trafficking proteins in plants [77], which supports the plausibility of similar effects on RAB3B, although this link remains to be explored. Our findings show that MOE treatment restores the altered expression and potentially the activity of RAB3B in brain slices subjected to OS. This suggests that MOE may protect vesicular trafficking mechanisms by preserving the normal function and regulation of RAB3B, thereby supporting neuronal resilience and function under stress conditions.

MIF emerged as one of the key proteins modulated by OS and restored by MOE treatment. MIF is a cytokine that plays a crucial role in regulating immune and inflammatory responses by promoting the production of pro-inflammatory mediators such as TNF-α, IL-1, and IL-6 [78]. MIF expression, particularly high in brain, bone marrow, reproductive, and lymphoid tissues, has been shown to increase under OS conditions, and growing evidence suggests that antioxidants may modulate its activity [79]. In rat models, OS has been associated with altered MIF levels, supporting the hypothesis that antioxidant-based interventions could reduce inflammation through MIF regulation. In this context, MOE may act as one such antioxidant, potentially contributing to the downregulation of MIF and the mitigation of OS-induced inflammatory responses in brain slices. Notably, MIF has also been proposed as a potential biomarker and therapeutic target for various neurological diseases, including AD and PD [79]. Together, these results highlight the multifaceted molecular effects of MOE in counteracting OS-induced proteomic alterations, supporting its potential as a multitarget neuroprotective agent.

## 4. Materials and Methods

### 4.1. Plant Materials and Solutions

*Magnolia officinalis* (L.) bark extract (MOE) was supplied by FAGRON (Quarto Inferiore, Italy, https://www.fagron.it/, accessed on 27 March 2026). Analytical separations for the quantitative analysis of the phytomarkers (magnolol and honokiol) were carried out by using an HPLC equipped with a PU-1580 pump and a diode-array detector (DAD) model MD-910 using the integration program Borwin-PDA (Jasco Corporation, Tokyo, Japan). A Kinetex^®^ F5 column (150 × 4.6 mm; 5 µm, Phenomenex SrL Castel Maggiore, Bologna, Italy) was used in the isocratic mode with a mobile phase composed of aqueous formic acid (0.4%, *v*/*v*) and acetonitrile, 45/55 (*v*/*v*), at a flow rate of 1 mL/min. Detection was performed at 250 nm. Details about sample preparation and the quantification were already reported [11]. The quality control of commercial magnolia bark extracts is based on the quantification of honokiol and magnolol, as these are reported as the main responsible active compounds for the beneficial properties of magnolia bark extract [80,81,82], and their amount is taken as a suitable attribute for the quality assurance and standardization of *Magnolia officinalis* (L.) bark extract samples [83]. The applied HPLC-UV DAD method allowed for a fast and selective separation of the two reference compounds: honokiol was contained at 29.1 ± 1.39 mg/g (*n* = 3) and magnolol at 16.2 ± 0.81 mg/g (*n* = 3) [11].

A 50 mg/mL MOE stock solution was freshly prepared immediately before use by solubilizing the powder in EtOH. From this, several dilutions were obtained by using artificial cerebrospinal fluid (ACSF, see below for the composition). The final concentration of ethanol was in the range of 0.01% (MOE 1 µg/mL) to 0.25% (MOE 25 µg/mL). Appropriate controls with ethanol were consistently conducted in parallel, demonstrating no interference with the measured parameter. The stability of the MOE solutions kept at different temperatures and times was checked by recording UV−Vis spectra in the wavelength range of 200–600 nm in a quartz cuvette with a 1 cm optical path length (Multiskan TM GO, Thermo Fisher Scientific, Vantaa, Finland). No significant changes were detected in the spectra of solutions kept at 37 °C for up to 24 h (see Supplementary Data, Figure S4), thus suggesting good stability of MOE during the treatment of brain slices (see below).

### 4.2. DPPH Assay

The free radical scavenging activity of MOE was assessed using a colorimetric DPPH (2,2-diphenyl-1-picrylhydrazyl) assay, following the procedure described in a previous study [84]. A DPPH stock solution (Merck KGaA, Darmstadt, Germany) was prepared by dissolving the compound in 99.8% methanol to reach a final concentration of 1 × 10^−4^ M. Ascorbic acid (Merck KGaA, Darmstadt, Germany) served as the standard antioxidant. All measurements were conducted in triplicate. The radical scavenging effect was expressed as percentage inhibition, calculated using the following formula: % inhibition = (Absc − Absx)/Absc × 100, where Absc is the absorbance of the positive control and Absx the absorbance of the tested samples. The IC_50_ value, indicating the concentration required to inhibit 50% of the DPPH radicals, was determined by plotting the inhibition percentages against the corresponding concentrations and fitting the data to a sigmoidal curve.

### 4.3. Rat Brain Slices

**Figure 10:**
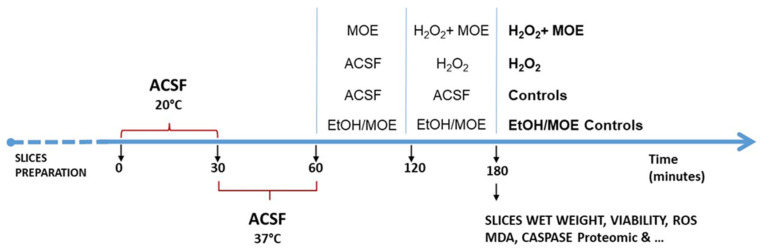
Neuroprotective effects of *Magnolia officinalis* (L.) extract (MOE) toward hydrogen peroxide-induced injury in rat brain cortical slices: experimental protocols used.

The Animal Care and experimental protocols conformed to the European Union Guidelines for the Care and the Use of Laboratory Animals (European Union Directive 2010/63/EU) and were approved by the Italian Department of Health (7DF19.N.TBT). The experimental protocol has been previously detailed [85]. Briefly, 400 μm thick cortical slices from male Wistar rats (Charles River, Calco, Italy) were immersed in artificial cerebrospinal fluid (ACSF; composition in mM: 120 NaCl, 2 KCl, 1 CaCl_2_, 1 MgSO_4_, 25 HEPES, 1 KH_2_PO_4_, and 10 glucose; final pH 7.4) and continuously aerated with a gas mixture of 95% O_2_ and 5% CO_2_. All reagents were purchased from Merck KGaA (Darmstadt, Germany). The slices were then allowed to recover at room temperature for 30 min and were subsequently moved to 24-well culture plates so that each well contained 2–3 slices (average weight ~30–40 mg in 0.5 mL ACSF at 37 °C). These were then incubated for another 30 min, changing the medium with fresh, oxygenated ACSF every 15 min (Figure 10).

After the equilibration phase at 37 °C, the slices were incubated with ACSF containing (or not, in the case of the controls) MOE (0.001–200 µg/mL in ACSF) for 1 h. After this period, the medium with MOE was maintained, and H_2_O_2_ (20 mM for 1 h, which causes about 50% tissue death) [85] was added. At the end of the treatments, the slices were used for assessing tissue viability, ROS, and MDA levels or treated as reported below for proteomic analyses. Finally, the effects of MOE per se were assessed by treating slices with MOE at concentrations of 10–200 μg/mL for 2 h, after which an MTT assay was performed.

### 4.4. Viability Assays

MTT (3-(4,5-dimethylthiazol-2-yl)-2,5-diphenyltetrazolium bromide) assay was employed to evaluate slice viability, as already described [85]. In some experiments, slices were fixed by immersion in 2 mL of 4% formalin for 24 h in the dark. After gently drying with paper, photographs were taken, and the areas of injury identified by reduced MTT staining vs. total area were calculated by an expert blind operator using the ImageJ software (version 1.54g, National Institute of Health, Bethesda, MD, USA) [85].

### 4.5. ROS, Lipid Peroxidation, and Caspase-3, -8, and -9 Activity

After the pretreatment with MOE and before the H_2_O_2_ treatment, slices were loaded with ACSF containing DCFH-DA (20 µM, 10 min in the dark). At the end of the experiments, the slices were carefully washed with cold PBS and homogenized in 500 µL of PBS. The fluorescence was measured using wavelengths of excitation and emission of 480 and 520 nm, respectively (Synergy HTX multi-mode reader, BioTek, Winooski, VT, USA), and normalized to the content of proteins. Average values in the control slices were taken as 100%. Lipid peroxidation was assessed by measuring malondialdehyde (MDA) levels, a key byproduct of lipid peroxidation, using the thiobarbituric acid-reactive substances (TBARS) assay, which quantifies MDA and provides an indirect measure of oxidative damage to cell membranes, as previously described [85].

For caspase activity, the protocol reported in [86] was used. Brain slices (~50 mg) were added to cold caspase lysis buffer (200 µL, composition: 20 mM HEPES/KOH, 10 mM KCl, 1.5 mM MgCl_2_, 1 mM EGTA, 1 mM EDTA, 1.1 mM DTT, 1 mM PMSF, and 10 μg/mL leupeptin; pH 7.5), homogenated, and centrifuged (12.000 g, 10 min at 4 °C). The supernatants were then kept on ice for an immediate assay or stored at −80 °C. For the assay, cell lysates (20–100 µg proteins) were incubated (1 h, 37 °C) with the fluorogenic substrates DEVD-AMC (Ac-Asp-Glu-Val-Asp-7-amino-4-methylcoumarin for caspase-3), IETD-AMC (Ac-Ile-Glu-Thr-Asp-AMC for caspase-8), or LEHD-AMC (Ac-Leu-Glu-His-Asp-7-amino-4-methylcoumarin for caspase-9) (PeptaNova GmbH, Sandhausen, Germany). These were used at 20 μM in 0.25 mL of caspase assay buffer (25 mM HEPES, 0.1% *w*/*v* CHAPS, 10% *w*/*v* sucrose, 10 mM DTT, and 0.01% *w*/*v* albumin serum bovine; pH 7.5). The reaction was stopped by adding 0.1% *w*/*v* ice-cold trichloroacetic acid (0.75 mL), and the fluorescence of the AMC (7-amino-4-methylcoumarin) fragment released by active caspases was then read (Fluoroskan Ascent fluorimeter, Thermo LabSystems, Waltham, MA, USA; 380 nm excitation and 460 nm emission wavelengths).

### 4.6. Proteomics Analysis

For the proteomic analysis, brain slices were lysed in ice-cold buffer containing 8 M urea, 2% (*w*/*v*) SDS, 100 mM Tris-HCl, pH 8, and a protease inhibitor cocktail and stored at −80 °C until use (*n* = 5 for each condition tested) [87]. A quality control (QC) sample was prepared by mixing equal amounts of protein extracts derived from brain slices and divided into 3 independent aliquots. For the analysis, lysates of brain slices and QC samples were processed following the filter-aided sample preparation (FASP) method [88], as already reported in [85,89]. Briefly, 50 µg of total proteins was diluted in 200 µL of 8 M urea in 50 mM Tris-HCl, pH 8, loaded into filters with a molecular weight cut-off of 10 kDa (Sartorius, Goettingen, Germany), and centrifuged (15 min, 14,000 g, 25 °C). Reduction of disulfide bonds was performed by adding 200 µL of 25 mM DTT and 50 mM Tris-HCl (45 min at 55 °C), and after centrifugation, alkylation of cysteines occurred by incubating filters at an equal volume of 50 mM Tris-HCl containing 55 mM iodoacetamide (45 min in the dark at RT). After centrifugation (15 min, 14,000 g, 25 °C), filters were incubated (18 h, 37 °C) in the presence of 2 µg of sequencing-grade modified trypsin (Promega, Madison, WI, USA) solution (100 μL in 50 mM Tris-HCl, 1 mM CaCl_2_; pH 8) to a final enzyme:protein ratio of 1:25 (*w*/*w*). Filters were centrifuged (10 min, 14,000 g), rinsed with 50 µL of 50 mM Tris-HCl, pH 8, and centrifuged again (10 min, 14,000 g) to collect digested peptides. To proceed with the MS analysis, peptides were purified using C_18_ BioPure spin columns, dried using a stream of nitrogen at room temperature, and, finally, suspended in 100 μL of 95/5 H_2_O/acetonitrile (*v*/*v*) containing 0.1% formic acid (*v*/*v*).

#### 4.6.1. Non-Targeted LC-HRMS/MS Analysis

Analysis of brain slice proteomes was performed using a hybrid quadrupole-Orbitrap Q-Exactive (Thermo Fisher Scientific, Waltham, MA, USA) mass spectrometer, coupled with an Ultimate 3000 UHPLC system (Thermo Fisher Scientific, Waltham, MA, USA). A total of 5 µg of digested proteins (10 µL) was loaded and separated on a reversed-phase analytical column (Aeris peptide C_18_, 150 × 2.1 mm, 2.6 μm, Phenomenex, Torrance, CA, USA) and kept at 30 °C. The chromatographic separation of peptides was carried out at a flow rate of 200 μL/min using water (A) and acetonitrile (B), both containing 0.1% formic acid (*v*/*v*), as mobile phases. The following gradient was used (expressed as an A:B ratio): for 1 min; from 97.5:2.5 to 30:70 from 1 to 20 min (following a linear gradient), then linearly changed from 30:7o to 50:50 in 4 min and changed again linearly to 5:95 in 2 min and from 50:50 to 20:80 in 2 min; 20:80 for 5 min and kept unchanged until 30 min. The system was re-equilibrated to the initial condition in 0.5 min and maintained until the end of the chromatographic run (35 min). The instrumental conditions were as previously described [85]. Data were acquired in the full-scan acquisition mode with data-dependent fragmentation of the four most intense ions. Full-scan spectra were acquired in a mass range between 300 and 2000 *m*/*z* at a resolving power of 70,000 FWHM at 200 *m*/*z*.

MS/MS spectra were acquired at a resolving power of 17,500 FWHM at 200 *m*/*z*, using an isolation window of 1.6 *m*/*z*, a normalized collision energy fixed to 27, and a dynamic exclusion of 30 s. The capillary temperature and heater temperature were set to 325 °C, with an applied voltage of 3.0 kV in positive polarity electrospray ionization. The sheath gas and auxiliary gas flow rates were 25 and 10 arbitrary units, respectively. A static exclusion list (including peptides discovered in the first run) was applied to the second LC-HRMS/MS analysis of each sample to increase the number of detected peptides.

#### 4.6.2. Non-Targeted MS Data Analysis

MS raw files were analyzed with Proteome Discoverer 2.1 software (Thermo Fisher Scientific, Waltham, MA, USA) using SEQUEST HT as the search engine (Thermo Fisher Scientific, Waltham, MA, USA). Spectra were searched against the UniProtKB *Rattus Norvegicus* database (UP000002494), including a decoy database. Trypsin was selected as the enzyme with up to 2 missed cleavages allowed. Carbamidomethyl-cysteine was set as a fixed modification, while methionine oxidation and pyro-glutamic conversion at the N-terminus were set as variable modifications. Peptide and fragment mass tolerances were set at 10 ppm and 0.1 Da, respectively. The principle of maximum parsimony was applied to group proteins into protein families. The percolator algorithm was used to assess the confidence of peptide and protein identification, allowing for a maximum false discovery rate of 1%. Only unique peptides were considered for label-free relative quantification, which was performed directly by the software by using the precursor area detector function. The mass spectrometry proteomics data were deposited to the ProteomeXchange Consortium via the PRIDE [90] partner repository with the dataset identifier PXD075711. We finally exported quantitative data from a spreadsheet for statistical analyses.

#### 4.6.3. Targeted PRM Analysis

We developed a scheduled parallel reaction monitoring (PRM) method using the Skyline software, version 3.5.0.9191 (MacCoss lab software) [91], for the targeted detection of peptides coming from proteins resulting as potentially altered by H_2_O_2_ treatment and reverted to control conditions by MOE. Acquisition was achieved with the same instruments and chromatographic method used for the non-targeted proteomics experiment, but HRMS/MS spectra were acquired in the PRM mode at 35,000 FWHM at 200 *m*/*z* using an inclusion list containing the *m*/*z* value of the target peptides (see Supplementary Data, Table S5). Protein digests undergoing the non-targeted experiments were analyzed again using the PRM method in a different analytical session. We injected repeatedly one of the QC samples during the analytical session to ensure that no instrumental drift occurred during acquisition and to normalize relative quantification data. Raw files were processed using the Skyline software, and relative quantification was achieved by integrating and summing the peak area of four precursor-to-product ion transitions for each peptide. Signal intensity for a given peptide was normalized to the signal intensity of the same peptide in the QC sample to obtain values of the same order of magnitude.

#### 4.6.4. Promoters Analysis

Promoters for all the genes were retrieved by using the Eukaryotic Promoter Database (EPD), considering 100 bp downstream the transcription start site (TSS) and 900 bp upstream the TSS (Supplementary Table S2). As a control, the same number of promoter sequences presenting random sequences were used. They were generated using the RANDNA tool [92].

To identify possible Transcription Factor Binding Sites (TFBSs) on the promoter sequences, the Match 1.0 algorithm [93] was used. Match is a weight matrix-based program for predicting TFBSs in DNA sequences using a library of positional weight matrices from the TRANSFAC^®^ [93] Public 6.0 database. Only TRANSFAC matrices derived from vertebrate TFBSs were used for the analyses of our promoter sequences. In our analyses, were minimized both false and positive results. The F-Match algorithm was used to identify statistically over-represented TFBSs in the set of promoter sequences compared to the control set. Statistical significance was considered when the *p*-value resulted lower than 0.01.

### 4.7. Analysis of Data

#### 4.7.1. Slices

The results were reported as means ± SEMs, and *n* represents the number of independent experiments (generally run in 4–6 replicates). The statistical significance was assessed by using a one-way ANOVA, followed by Bonferroni post hoc tests (GraphPad Prism version 5.04, GraphPad Software Inc., San Diego, CA, USA). In all comparisons, the level of statistical significance (*p*) was set to 0.05.

#### 4.7.2. Proteomic Analysis

For the proteomic analysis, quantitative data were examined by a principal component analysis (PCA) using the SIMCA-P software (version 13.0, Umetrics, Umeå, Sweden). This analysis visualized the distribution of samples based on the proteomic profile of the analyzed samples. A one-way ANOVA was applied to compare protein abundance of the control, H_2_O_2_-treated, and H_2_O_2_/MOE samples. Fold changes for the quantified proteins were calculated as the ratio of protein abundance in the H_2_O_2_-treated samples relative to controls (H_2_O_2_/control) and in the H_2_O_2_-treated samples relative to the MOE-treated samples (H_2_O_2_/MOE). To identify proteins significantly affected by the H_2_O_2_ treatment, we applied selection criteria, both based on fold change and statistical significance. Specifically, we considered altered proteins with expression values higher than 1.25 or lower than 0.8 (H_2_O_2_/control) and a *p*-value < 0.05. A volcano plot was used to visualize the relationship between fold change and statistical significance. Among the proteins whose expression was affected by the H_2_O_2_ treatment, we focused on those whose expression was restored after the MOE treatment. Indeed, this expression pattern indicates a normalization effect of MOE on protein expression that has been affected by OS. The results were reported as mean ± SEM.

#### 4.7.3. Bioinformatic Analysis

The EnrichR webtool, using default parameters (Ma’ayan Lab, Icahn School of Medicine at Mount Sinai, New York, NY, USA [94]), was used to perform a gene ontology (GO) enrichment analysis. The MeV software (version 4.9.0, SourceForge, San Diego, CA, USA) was used to perform a cluster analysis. The publicly accessible Cytoscape 3.10.2 application integrated with the STRING tool [95] was used to conduct the protein–protein interaction network and gene ontology enrichment analysis by combining a variety of prediction approaches with additional data (e.g., neighborhood, transferred neighborhood, gene fusion, co-occurrence, co-expression, experimental data, databases, and text mining).

The protein network was constructed using default parameters (minimum required interaction score = 0.4) with all active prediction parameters.

## 5. Conclusions

The research of effective treatments for NDs represents a significant challenge. Natural products from plants constitute an important source of neuroprotective agents by acting simultaneously on multiple targets. The understanding of their activity might drive the research toward the discovery of novel drugs for delaying the onset or the progression of these diseases [96,97,98]. The intake of phytochemicals on a regular basis might also boost the antioxidant system, thus increasing neuronal cell survival and improving physical and mental activity [99]. The present findings highlight the interesting neuroprotective properties of MOE, which prevented the formation of ROS and lipid peroxidation, as well as the changes in the activation of the main proteins involved in apoptosis in rat brain slices. Moreover, the proteomic analysis revealed that MOE effectively counteracts protein alterations induced by OS, restoring the levels of 14 proteins whose expression was significantly affected by H_2_O_2_, suggesting a key role in proteostasis and neuroprotection. Among the most relevant proteins, VCP and calnexin are crucial for cellular stress responses and protein homeostasis, while α-synuclein and Psmb2 are directly implicated in neurodegeneration. The restoration of their expression in an ex vivo model suggests that MOE may act by modulating protein metabolism and preventing the accumulation of misfolded or damaged proteins, a hallmark of NDs. The present study indicates that MOE activity extends beyond simply reducing OS, as it also plays a broader role in regulating protein degradation pathways, inflammatory responses, and neuronal survival mechanisms. However, further studies are necessary to elucidate the molecular mechanisms underlying these proteomic modulations and to determine whether MOE’s neuroprotective effects persist in in vivo models of neurodegeneration. Finally, the available literature supports a favorable safety profile of MOE extract in vivo, including the absence of genotoxic effects in standard micronucleus assays [100], and several in vivo studies have demonstrated neuroprotective and anti-inflammatory effects of its main bioactive constituents (e.g., honokiol and magnolol) in rodent models, including attenuation of neuropathic pain and modulation of neuroinflammatory pathways [36,101]. These findings support the biological plausibility of sustained protective effects under longer exposure conditions.

## Figures and Tables

**Table 1:** List of 33 proteins significantly affected by H_2_O_2_ treatment. The abundance ratios and the ANOVA *p*-values allow for a comparison among protein amounts observed in the non-targeted proteomics analysis for the different conditions studied. Proteins highlighted in light blue are the ones whose amounts were affected by H_2_O_2_ treatment but counteracted by the addition of MOE (5 µg/mL). * Proteins confirmed by PRM experiment, as affected by H_2_O_2_ treatment and maintained to control conditions by MOE addition.

		Abundance Ratio			ANOVA *p*-Value		
Protein Accession (UniProtKB)	Description	H_2_O_2_/Control	H_2_O_2_/(MOE + H_2_O_2_)	Control/(MOE + H_2_O_2_)	H_2_O_2_ vs. Control	H_2_O_2_ vs. (MOE + H_2_O_2_)	Control vs. (MOE + H_2_O_2_)
sp|P46462|TERA_RAT *	Transitional endoplasmic reticulum ATPase	1.57	1.60	1.02	0.029	0.025	0.942
sp|P37377|SYUA_RAT *	Alpha-synuclein	1.46	1.45	0.99	0.025	0.028	0.949
sp|P62138|PP1A_RAT *	Serine/threonine-protein phosphatase PP1-alpha catalytic subunit	1.37	1.51	1.10	0.045	0.018	0.614
sp|P35565|CALX_RAT *	Calnexin	1.46	1.62	1.11	0.022	0.007	0.576
sp|Q63941|RAB3B_RAT *	Ras-related protein Rab-3B	1.54	1.54	1.00	0.045	0.044	0.995
sp|P55161|NCKP1_RAT *	Nck-associated protein 1	2.16	1.61	0.74	0.036	0.044	0.809
sp|P04692|TPM1_RAT *	Tropomyosin alpha-1 chain	1.27	1.62	1.27	0.037	0.001	0.090
sp|P17764|THIL_RAT *	Acetyl-CoA acetyltransferase, mitochondrial	1.38	1.57	1.14	0.047	0.013	0.493
tr|B2GV99|B2GV99_RAT *	Myl6 protein	1.55	1.54	1.00	0.016	0.017	0.981
sp|P0C5X8|TTYH1_RAT *	Protein tweety homolog 1	1.83	1.53	0.84	0.013	0.045	0.514
sp|P86252|PURA_RAT *	Transcriptional activator protein Pur-alpha	1.66	1.59	0.96	0.006	0.009	0.831
sp|B2RYG6|OTUB1_RAT *	Ubiquitin thioesterase OTUB1	1.52	1.78	1.17	0.016	0.004	0.448
sp|P04906|GSTP1_RAT	Glutathione S-transferase	1.60	1.98	1.24	0.036	0.009	0.454
sp|P11661|NU5M_RAT	NADH-ubiquinone oxidoreductase chain 5	1.52	1.44	0.95	0.008	0.015	0.738
sp|F1LQ48|HNRPL_RAT	Heterogeneous nuclear ribonucleoprotein L	4.04	1.85	0.46	0.014	0.023	0.530
sp|P30904|MIF_RAT *	Macrophage migration inhibitory factor	1.45	1.48	1.02	0.043	0.035	0.914
sp|Q6PEC4|SKP1_RAT	S-phase kinase-associated protein 1	1.26	1.39	1.11	0.023	0.004	0.357
sp|P40307|PSB2_RAT *	Proteasome subunit beta type-2	1.77	1.75	0.99	0.026	0.029	0.966
sp|Q63327|MOBP_RAT	Myelin-associated oligodendrocyte basic protein	0.78	1.06	1.37	0.009	0.512	0.004
sp|P85969|SNAB_RAT	Beta-soluble NSF attachment protein	1.57	1.37	0.87	0.013	0.050	0.472
sp|P12369|KAP3_RAT	cAMP-dependent protein kinase type II-beta regulatory subunit	1.95	1.28	0.66	0.023	0.266	0.173
tr|A0A0G2K654|A0A0G2K654_RAT	H1.2 linker histone, cluster member	1.30	1.12	0.87	0.024	0.237	0.203
sp|P05503|COX1_RAT	Cytochrome c oxidase subunit 1	1.88	1.37	0.73	0.030	0.178	0.321
tr|D3ZS58|D3ZS58_RAT	NADH dehydrogenase [ubiquinone] 1 alpha subcomplex subunit 2	1.63	1.30	0.79	0.032	0.177	0.338
sp|Q4FZY0|EFHD2_RAT	EF-hand domain-containing protein D2	1.38	1.26	0.91	0.033	0.095	0.530
sp|P81155|VDAC2_RAT	Voltage-dependent anion-selective channel protein 2	1.43	1.41	0.99	0.035	0.087	0.940
sp|Q9JK11|RTN4_RAT	Reticulon-4	1.49	1.29	0.87	0.037	0.131	0.481
sp|P62483|KCAB2_RAT	Voltage-gated potassium channel subunit beta-2	1.64	1.50	0.91	0.038	0.057	0.733
tr|A0A0G2K459|A0A0G2K459_RAT	Mitochondrial carrier 2	1.54	1.38	0.90	0.039	0.092	0.637
sp|P07895|SODM_RAT	Superoxide dismutase [Mn], mitochondrial	1.50	1.32	0.88	0.040	0.121	0.542
sp|Q3KR86|MIC60_RAT	MICOS complex subunit Mic60 (Fragment)	1.60	1.33	0.83	0.041	0.159	0.450
sp|A0JPJ7|OLA1_RAT	Obg-like ATPase 1	1.74	1.42	0.81	0.044	0.138	0.531
sp|Q08163|CAP1_RAT	Adenylyl cyclase-associated protein 1	1.79	1.58	0.88	0.045	0.087	0.710

**Table 2:** Proteins significantly affected by H_2_O_2_ treatment and reverted to control conditions by MOE treatment (5 µg/mL). Protein accession number, description, and mean abundance ratio among different conditions tested (i.e., control, H_2_O_2_, and H_2_O_2_ + MOE).

		Mean Abundance Ratio		
Protein Accession (UniProtKB)	Protein Description	H_2_O_2_/Control	H_2_O_2_/(MOE + H_2_O_2_)	Control/(MOE + H_2_O_2_)
sp|P46462|TERA_RAT	Transitional endoplasmic reticulum ATPase	1.57	1.60	1.02
sp|P37377|SYUA_RAT	Alpha-synuclein	1.46	1.45	0.99
sp|P62138|PP1A_RAT	Serine/threonine-protein phosphatase PP1-alpha catalytic subunit	1.37	1.51	1.10
sp|P35565|CALX_RAT	Calnexin	1.46	1.62	1.11
sp|Q63941|RAB3B_RAT	Ras-related protein Rab-3B	1.54	1.54	1.00
sp|P55161|NCKP1_RAT	Nck-associated protein 1	2.16	1.61	0.74
sp|P04692|TPM1_RAT	Tropomyosin alpha-1 chain	1.27	1.62	1.27
sp|P17764|THIL_RAT	Acetyl-CoA acetyltransferase, mitochondrial	1.38	1.57	1.14
tr|B2GV99|B2GV99_RAT	Myl6 protein	1.55	1.54	1.00
sp|P0C5X8|TTYH1_RAT	Protein tweety homolog 1	1.83	1.53	0.84
sp|P86252|PURA_RAT	Transcriptional activator protein Pur-alpha	1.66	1.59	0.96
sp|B2RYG6|OTUB1_RAT	Ubiquitin thioesterase OTUB1	1.52	1.78	1.17
sp|P04906|GSTP1_RAT	Glutathione S-transferase	1.60	1.98	1.24
sp|P11661|NU5M_RAT	NADH-ubiquinone oxidoreductase chain 5	1.52	1.44	0.95
sp|F1LQ48|HNRPL_RAT	Heterogeneous nuclear ribonucleoprotein L	4.04	1.85	0.46
sp|P30904|MIF_RAT	Macrophage migration inhibitory factor	1.45	1.48	1.02
sp|Q6PEC4|SKP1_RAT	S-phase kinase-associated protein 1	1.26	1.39	1.11
sp|P40307|PSB2_RAT	Proteasome subunit beta type-2	1.77	1.75	0.99

## Data Availability

Data will be made available on request.

## Supplementary Materials

The following supporting information can be downloaded at https://www.mdpi.com/article/10.3390/ijms27083350/s1.
