# Supplementary material for: Magnolia officinalis (L.) Bark Extract Counteracts Oxidative Brain Injury: A Proteomic Investigation into Neuroprotective Mechanisms
Source: Int J Mol Sci. 2026 Apr 8;27(8):3350. doi: 10.3390/ijms27083350 (PMC13116406; doi:10.3390/ijms27083350)
Supplement: Supplementary file 1 [file ijms-27-03350-s001.zip › ijms-4173096-supplementary/Supplementary IJMS rev1.pdf]

Supplementary Data

Figure S1: Effects of ethanol on rat brain slices viability

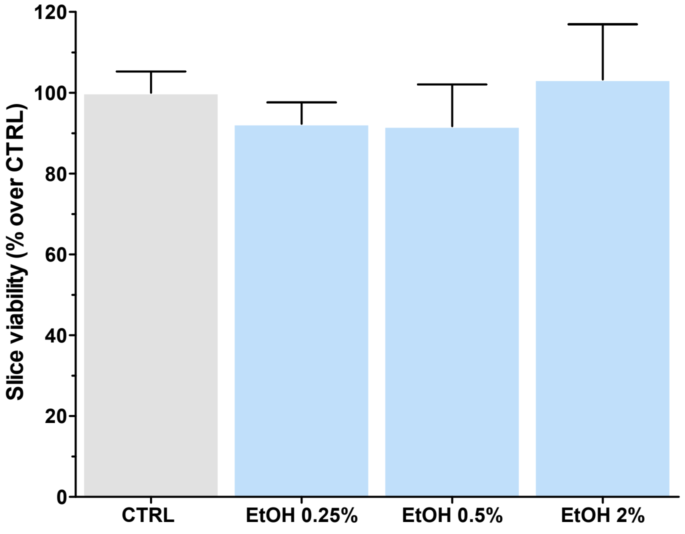

Figure S1: Effect of ethanol (0.25%-2% v/v) on the viability of rat brain slices after 2 hours of treatment. Data are shown as mean ± SEM (n=3-4); controls (CTRL) represent slices treated with ACSF.

Figure S2: Effects of MOE on prolonged oxidative stress

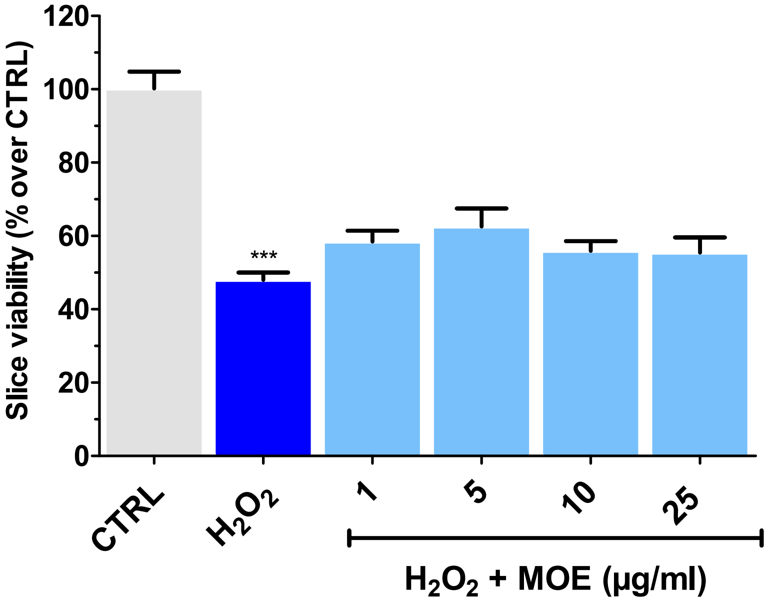

Figure S2: Effect of MOE (1-25 µg/ml) on prolonged oxidative stress- (H_2O_2 20 mM for 2h) induced injury, in rat brain slices. Data were reported as mean ± SEM (n=3-4). Statistical analysis was performed by ANOVA followed by Bonferroni post-hoc test. ***$P<0.001$ vs CTRL.

Figure S3: Antioxidant activity of MOE

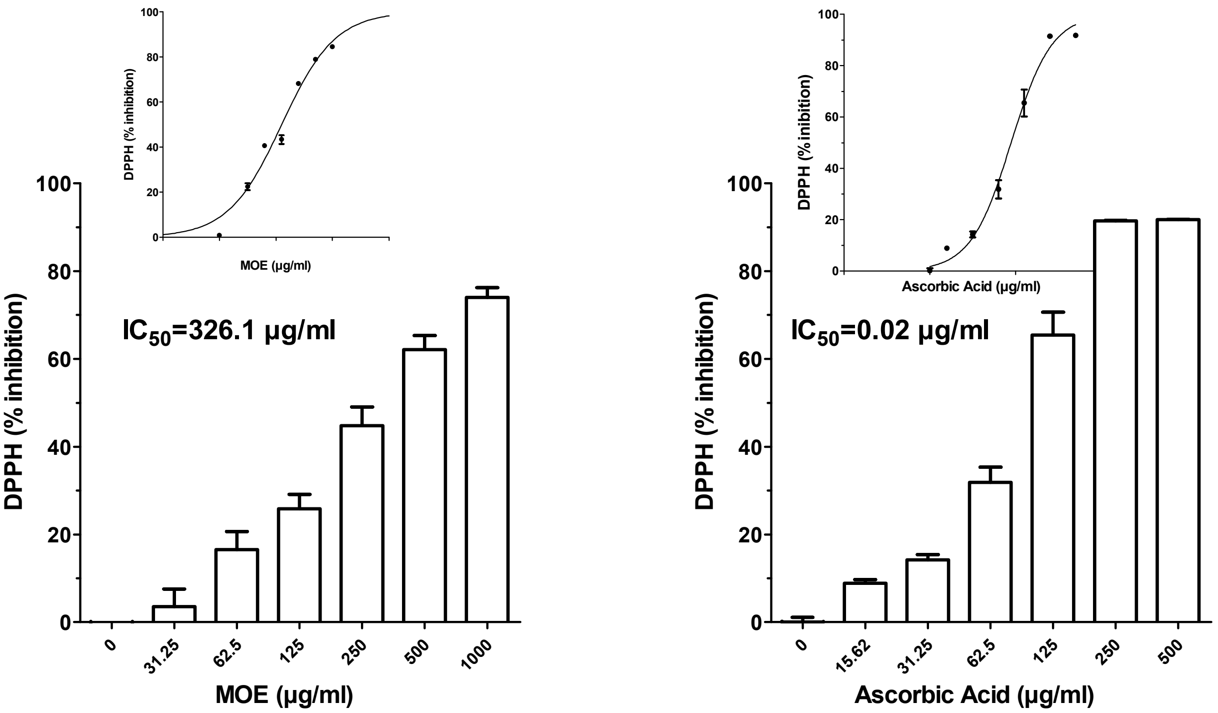

Figure S3: MOE (left) and Ascorbic acid (right) antioxidant activity as measured by DPPH assay. Absorbance was measured at 517 nm after 30 min of incubation with DPPH 100 µM. DPPH scavenged (%) is represented in Y-axis. Data are reported as mean ±SEM (n=4). IC_{50} values were calculated by fitting the data according to a sigmoidal curve (log inhibitor vs normalized response, see insert).

Figure S4: Stability of MOE solution

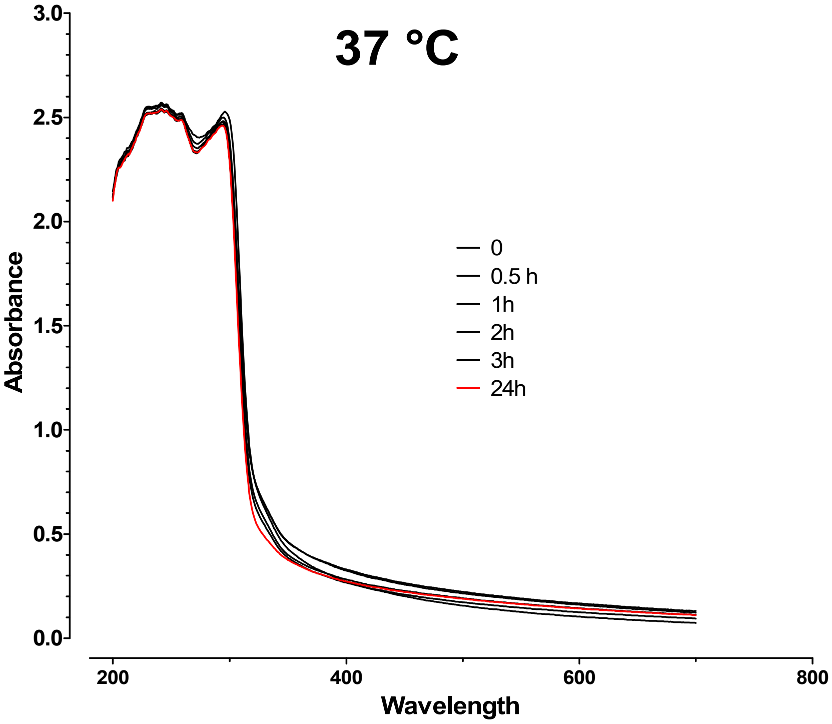

Figure S4: UV-visible spectra of MOE solutions (0.3 mg/ml) incubate at 37°C for different times. The stability of MOE solutions kept at different temperatures and times were checked by recording UV-Vis spectra in the wavelength range of 200-600 nm in quartz cuvette with 1 cm optical path length. No significant changes were detected in the spectra of solutions up to 24h.

Table S1. List of identified proteins in the proteomics experiment

See file excel

Table S2

Sequences of gene promoters retrieved by using EPD and control random sequences. When the gene may have more than one promoter all the promoters were considered (they can be distinguished by _1 and _2 in the Gene Name column). Uppercase is used to distinguish 100 bp downstream the TSS.

Gene Name	Ensembl	Promoter Sequence
Vcp	ENSRNOG00000034242	>FP004192 Vcp_1 :+U EU:NC; range -900 to 100.<br>ctggcttcaaactgtcaatctcctgcctttagcctctggagtactgagatctagggtgtacaccaccatacccacttg<br>tttttgcttgttttaaaattttatttacttagttttgagtatcatttggctggctggaactctttttataggccaggctga<br>cttaaaactcacagagagccgcctgcctccgaagtgctgacattaaatgcatgtactaccacactgcttttaaaatt<br>tattgagatactgtttgagactagaattatagctatgtgccattacgccgacatggttttgtttttcagaaaaggtctt<br>gtatcctgggttgatctccatctaatagtcctcctgcctcagcttcccaattgctaaaaatacagacctagcccatcc<br>accacgcccttcgttggagccagtgttttaaaggagcacgaaggattttagtatggtggagtaagaggatccctag<br>gattggcgagaaatgaactatgtggattattattacaactcagctaaactaataagggttcaagcctttgtgcgag<br>gttctcccagctgcttataaactaaggagccaatcaattggagccaagtagaggaaacctccttcggagggataa<br>agactctccttttaccagaggatctggcctattgcttttgtcgattggtcaagactccctatgcgtcgaggaagcgta<br>gtgctacggccaattgacgtggcgttactaggcgtgttgcagccttgaggcggggctaggccacaaacgaattttc<br>tgattggttatcgtctgtgggttgctggggagaggcacggagaggcgggcacgagtcctaaggcagtcgctgattg<br>gttgaggttagagcagctttccttccgatgattcggcttttctcggttcAGTCTCCGTGAAGCGTTTGCAGC<br>CGTCGTTTGATTAGTCGCCTCTCGCGGATTAGGAGCTAGCGTCTCCCGCCCGCCTGCCG<br>CCCCGGTGCCGCTGGGAGG
Snca	ENSRNOG00000008656	>FP003616 Snca_1 :+U EU:NC; range -900 to 100.<br>aagcctgtgcatccatctgcctgtctgctgggcttggaagaaaggtctgttagctggaactccatgcagccagaag<br>tcggaaaggtaagaggtgtgcaaagtctgccattaagtaggaagaaactgcctgcgatggtcccagagggtgaat<br>cccacaggagctaccttccaatcctgtaacagggttctcaatagtaagccactttttcaagtgcaaaaaagccttta<br>ggcagctggttttcgacggttggggtttatttattccttgctccacagatgggggaaaaaaaaatcagcgtctggca<br>gccgctgattggtggaaacgaaaatggtgatagtggagtgggaaagaggatttgctgagcctcccctgcctcctc<br>gacctgtaactcttccttagtcggctccccttcacacccagaagaacctttttagactccttcggggttaaaacaaat<br>ggaattctcaggctgtgtgaacaaaagcaacccgaagggtgtgtgctccctcccctcgcctggctccgcacacaga<br>tcatttcaggcggttcagtcctctggtgtccgcttcccggctcgtccggaggaggggggtcgcctagaggaaccga<br>gaacaggctgaggcagggaagaaggggatgagataggaaagaggccagctcaagttcagccacgataaaacc<br>gaaggccccctgaactcgaggtgaaactcaggctaccctctcttccctcctcctggggagcgtgctcccggtaccca<br>cagccctcacgcaccgccatcccgtcccctccctaggaaaacgagcgaaggcacgaggcaggcgaggggcggg<br>gagaggagctgacaaatcagctgcgggggcgacgtgaaggagccagggagccagagcgcccggcagcAGAC<br>GGCAGGAGACCAGCAGGTGCTCCCCCTGCCCTTGCCCCTCAGCCCAGAGCCTTTCACCC<br>CTCTTGCATTGAAATTAGATTGGGGAAAACAGGAGGAA
Ppp1ca	ENSRNOG00000018708	>FP001143 Ppp1ca_1 :+U EU:NC; range -900 to 100.<br>cactgtcctgggttttctttttgagagtgaaagggtacagtctgggaacaagggtctcacgtagcccaggcagga<br>gatgaactcactatgtagttgagcatgaccctgaacttctgatcctcctgtgtccagactcctgagtgctggaatttc<br>aggcactggaattacaggtttatgatacattgggttctttaatgtgttactgtttaaaaataatacctttccccccttttt<br>ctttctttactttccttccagagccggagataaaacctcaggccttgcacatgctagacaagtccaagcattctaca<br>aaggacttacaacaggaaggagataaaatagtattacatcacttaacgaaaaaaaaaacctgacgtagcaggc<br>accaaggcaacctggaccaaaacattgcttcgagttcagtagcaggtgttcatgggtctggccataaagaccgag<br>gtgaatgtgaggctctggaggtttggagctcactctcagtgtcattgagccttagttcctttttctgcacttcgaggaa<br>ttactttacagtgtttgagaagtcagcgattaacatgctcacaccacaatcccacgcagggctggggtgggaagag

		acttggaaagagaaaaacaatttctacagtacttccggttctaactcatcagaacgcagcagggcgattcctcctg<br>ggtggagccgacccaactccgccctacaaaccccgccctccacccctgccaggcctcgcccggccgcccgaggcc<br>ctccccggctgcagtgcttccggtccaggggaggcgctagagagcagggaactacggacgcggcagggcggggc<br>ggccggccgaagaggcggggcggcgggccgagggcgggctggggagggcggaaggagagccAGGCCGGA<br>AGGAGGCTGCAAGAGGGCGGGAGGCAGGAGAGGGCCCGGAGCTGGTGGGCCGGAG<br>CGGCGGCGCCGCCATGTCCGACAGCGAGAAGCTCAACC
Canx	ENSRNOG00000003343	>FP007678 Canx_1 :+U EU:NC; range -900 to 100.<br>atcaaatccttaactgcctttctcttggggtagcaggaacaaagctggtttcatatgtagttacatagatgataaaa<br>atctgtttctttctccagtagtcaaagctttgggtcaccaaacacaaccaggagcgatcaggattttcaaagacata<br>gccaaatccatagccacttcaatcttacagcgttttcaccatattgaaaaaggagtgggttacgaaatggctatttt<br>aaatctgcacatggcatcaccctgtcgctagagtatgtctgattttaacgcctgaaacagcacgggaacatgggaa<br>ggtgagttcatgttgcatgattgtgactctagatagtgttttgttaggtaaaaaggtcccaggttcagggcgatgact<br>ccgcaagacagtgtctgctgcccagagggatctcattcgctgcataagcacactggcctgacctctcaccatttctg<br>atgaagggcataggtcccagacaggattggaacagggtttctaactcaatgggtgggaaaaggtgtcttttggat<br>ggccaccaacacaccggccctgaggaaccaggtcgacccacgcgaggacgcacgccaaatccgcccgccctccg<br>gcccgcgcacccgcccgtttggtgacggccgccttctttcctcatacccaatcagcaaccacttcctgcctcactctt<br>gtccaatcatcacgctccacgaagaatagccctagggggcgtatgggaaagagctttgagcgatggccgtatcta<br>ccaatagattctcagatcttgcgcaaagggacgggagttgacgtttgttgtagccactcaggaacgaggggcgga<br>cgcggggttgggcttcgtgcggtggggctcgctcgcgcggcggcggtagccgaggcctcttAGTTCTGCGGC<br>ACGTGACGGTCGGGCCGCCTCTGCCGCTGTCTCCACTGCAGCACGGGGCCCGGTGTGC<br>GGGTGGGAGAAGGTGAGGGAGCCGCCAGTGGT
Rab3b	ENSRNOG00000008001	>FP004430 Rab3b_1 :+U EU:NC; range -900 to 100.<br>ggcaactcacaattgtctgtaactctagttacaccctcacacagacatatatgcaggcaaaacaccaatgtacatg<br>aaataaaaataaatcgttaagagaaataaagaaaggaaggaaggaaagaataagaacactagaccctaactgt<br>catcccagggacctcaggacaatggaaagtgaaatccacagaaagatttgaagtcgagggtgctatttacctcct<br>gaacctaccactcgtggaagatctgggacctgctaagcctcagacaagaacatttaccaccgcagctggcaatcc<br>ccatgctactgctgttaatggagacgcagtgagggttattttaacaaggtcggtggcatttcaaaaggttactcggg<br>cccctggtataaaggctggaaagagaggcaggaattggaattcttgcattggttcaggccagaagctgtgagtcct<br>gaaccaggatacttttgcagcgaaaaggaaagtaagaggaaggattgaatttgttcagatgggattaccagtctc<br>aaaggacatggaccatgaaggggtggtgggcggggcagaggataatacccagcttcctagctcagttttcacttt<br>gtagacaggttgtgctccctccttgagagtttatgggaatggagtaaaagcatttggaaatagcaaaatttgacgc<br>catcatcatctacatcatcgtcaaagccctctgaacaccctgtaggtgaatgtggacagatcatatagttacccaga<br>tatacagggtctgggggggttcccaagaagtcctggatttcctacacctgccaggaacggaggggcaggacggatc<br>gtacgtgggttccgcctagaggcggggcctggacccagcgggaacccaacccatcttctgccagagcctcAACC<br>CTCCGTAGCCGAGGTGGGAACCAGACCCGCCCCGCCTGCCTCTCGTCCACTACTGCCA<br>GGTGCAACAGTCCGGCTGCAGTGTGCCGCGAGCCAGCTC
Nckap1l	ENSRNOG00000036829	>FP006243 Nckap1l_1 :+U EU:NC; range -900 to 100.<br>tgtaagtttctttttaaaacattttgttttattgttgtagtatctaacaagtttccaagtactagtgcttctgttgggtca<br>tgctcacagatcgatacgttcaatactactaaggatatcaatattagatatcatacctgtcattaggaagtaggaat<br>gatatggatgggggagagagaaggcataggcttaaaaatgtaacaaggaaggtaatcccagcattctggaggta<br>gaaagtaggagatcagaggttcaagtttatccccaactacataaagagatcaagtatagcctggggtttacaaga<br>ctctttttcaaccctcacaaccgtgtgtgtgtgtgtgtgtgtgtgtgtaataaatatacacatatatacacataaat<br>atgtttatgttttatgtttatacataaaatagagtgaatatgtcactgaatggaatagttagaaagagatgagggtt<br>ctaaagaatgctggggcccaagggtcagtagagaataacagatattcttgattggtatataaaggaaagtagagg<br>tcgggggcggggctggtagtagatattcatttgttgtgtgctttgtctgggacatttggggagggggaaggccaga<br>acggttggtgctgcattgtgaggaggcttgaaagcctgctcctttcctcggcttccgcccacttcttaccaaagtact<br>gtacaaccagaaagaggaagtctcctctgctgagggtgcaagaggaagcacaagagggctctgaaggcctatga<br>gccctcagccaataacagaagcaaccggaagcctggtgcaccaaccaggctgggcccttctacacagagggtgtt

		gtccaggctgagaatatttggggcaattgggtcttgctatccggatggtcttttttctCGGTGGCCATCATGT<br>CTTTGACCTCTGCTTACCAGCATAAGTTAGCAGAAAAGCTCACGATCCTGAATGACCGG<br>GGTCAAGGAGTTCTCATTCGCATGTAC
Tpm 1_1	ENSR NOG 0000 0018 184	>FP006620 Tpm1_1 :+U EU:NC; range -900 to 100.<br>cataactcagcaaaccacacacacacacacacacacacacacacacacacacacacacacacaccttggcaca<br>ctccttctaccctttccaaagcacagcacaggcacataactcagcaaaccgggccctaagttcctaatgtattttac<br>tgcccacctccagcttggtgaaacgattggagacttcctgtgtgtatcattatgcccaccatcacgggcagagaga<br>cagggcctcaggaatggcccccacaccttcctccacctctatttctctgcgtgggttgaaatttccagactgtgaga<br>gcccaggactatgactggtgggtggactcagagctgctagaaggacgcagctaggtactgagaactcccaggccc<br>agatgcaaggtgcggtggctgtgactggcagcccccggaccttacagtgagcaccccaggacccggaggccaag<br>tgagatggtgcgcacacaccacctgccctgttcccgggattccctgtgactcccgacagactcttctggggaccctg<br>accgccaggcgacggctctgggaggtggctagagactctcgcccagtaccgggctcctctccagcgaggtccggg<br>agctctttcccaggcgtgcaggtttagaaataggacggcctcgcccggccgcggtctcggaggctcgggtccccat<br>atatagtcatatccaccgtcaactgggaggccggcaggcggcagcgaatgggcgtgaggcccccgcgggaggag<br>cgaggaggggggcatggggcggaggcaggagactgggaaaggggggaaggaggaacaagcttttccaaaaaagt<br>attggctgtcctaaggaatgcggtcgccccccctgggaaagtacatatcagggagcagcaggcagctccgcgctcG<br>CACTCCGGCTCTGCCACCCTACCGCGCTCGCGTCTCCTCTGGCTCCTCTGCCTAAGGGC<br>CCCTCGCCACCGCCACCATGGACGCCATCAAGAAGAAGATG
Tpm 1_2	ENSR NOG 0000 0018 184	>FP006619 Tpm1_2 :+U EU:NC; range -900 to 100.<br>tggggtacagacaaaatcttatggtagcgtttaattacatagtttgagttcttaaataagtgaggcatatgtaaatg<br>ttttatatcatctgataacttcaacagccttgtgaagtgtcctgtactattatactcgtttttaagagaggaaatccct<br>gagaagtcatttgtccaaaggcacacagccagcagagacttaagagccagattgaaaccttaaaaaagatgtatt<br>tagaacaggagatttaaccactacgctttgccagctagtagtttacacttggtgagatagtttgggtgttgggattct<br>tgtagtgaggatcttgtgttctgtgttcttcacaaggatctttaggatattcctatttcaggctccgagttccttgttca<br>gactctgggaaatcgggagttgccaatgcctttagtattcggaatgatggcgttctttacaactcaaagagtttagg<br>gaacgttcaaggtctaggttcctacccttactcagcttcaggggaatctcgatttcagagcgctccaaatggccagc<br>aggagtcgccataggctttagcatccagttcctctacttggagtcacccaggctccgcggaatggatgcaaacgcc<br>ctcctgcggtagcctcgctctaggcaaagcagcccagagccagccattcccgcgcgcacgcgcagtggttccaag<br>ggtagccagttcctcgggtccaccgggccccgcccgcctgcccgccgcgcccatcccctgcgttcgcgcacccgcc<br>cgcggctctctagtgcggccctggggccgtgacttccgggctgcttcttagccgcagggggcgccggcgttgcaaa<br>gcgggcctgggcgggacgaaccggacctgggctccgtaactgcaggagcccagcAGTAGCAGCTGCAGA<br>GCGCGGAGGCAGAGCACAGCCTCAGCCGCCGCCTCGCCATGGCGGGTAGCAGCTCGC<br>TGGAGGCGGTGCGGAGAAAGATCCGGAGC
Acat 1	ENSR NOG 0000 0007 862	>FP006496 Acat1_1 :+U EU:NC; range -900 to 100.<br>aaagaaaattaatgttacaagtcagatctgctagccccaactgaagattaaaagtcagctaatgggctaggtata<br>gtgcttttaatcccagcactagggaggcaaagacagacagatctctgaatttaaggccatcctgatctacatagtg<br>aatcctagcatagccagactacacagtgggaccctatttaaaaactaaccaaacaaaacccaaacaaaacaacc<br>gcccaactaaccttagcaatgggttccacaaataggaaaattatcatttaactaatatcagttaatgcatagaggg<br>tttttgtttcgttttaattttttggagacaaggtctccgtttgtggttttagtgatttggggactcgctatgtggaccagg<br>ctggcctcgaactcatagcgctccacctacctctttcctgagtgtgtcctaccacacgcagcctacagggagtttctc<br>tgaaagttatgtaatcttattactcagtgaagtttagatagtaatatgtattttacttagcatcctacagaaaaacaa<br>cttcaaaattctcttaacatagtaattggtttatttgtattataaataaatgtaactggaattgctggatacgtttacc<br>aaaatcctttgtctggatagatacagtcacacggtcaggcgcagcagagtgaatggaaagtctgggactgtatgg<br>aaacaacaggcatcaagagcttaaaagcggtttaggctgcaccagccaggtgtcaagcctcgtggtcattagtcc<br>atttttgtccaggagagcctctcctctgtcctcctgccaagcgccaggccccgcccctggctacgggccaatcgccg<br>ctgagagcagcgatctctgactgctattggaggagagggccggggcggtgcccgccACGACGTCGTGGGG<br>TCTGCTGTGGGAAGTGGAAGCAGCTGCGCGTCTCTCTGCCCTTGACAAGCTTTTCCGGT<br>CTCCATGGCTGCCCTGGCGGTTCTACAC

Myl6	ENSRNOG00000054140	>FP005464 Myl6_1 :+U EU:NC; range -900 to 100.<br>ccaaggaactgttgataagtgccatacagaaagacgacagggagggtgggaggacgaaccaccctgagagtgg<br>cttggactagtgagtacgggaaggcagaggctctaactgtgtttgggttcaggagagaagatgactgaggaagag<br>gtagagactgttttggcaggccacgaggacagcaacggctgcatcaactatgagggtgagggggcaggatgacc<br>agggggggtgaggagtcttgagggggttgggagtgtaccctcgtttactcaaaggtcagtttctgaagtcctctcctc<br>tcctctcgtgtgtgcagccttcctgaagcacatcctaagcctctgagctccgcagcccctagtgccctctagagagg<br>caacgatcccggtcaaccagacaagcgcaggcagtgcgtttccccatccgactgtagcgcagccgccagcgctgg<br>acaactttctttctaccccacccaactccaccccaccccacgtagaacctgcgggatcggaaacttgccccacctcc<br>tgtgcggccagtagtgcgttcctgccactaggggcacttacggttttgaaataaaaacatggttcctgctttggttt<br>ccgactggtttcttttgaggaaggggaaggacgggagcggtagtcctttcttctgcaaaagtccaaatcccctgctc<br>ttagtgcagttcggaagaaaggcttccaagagtcacaccctaggcctccgccgtgctcctgagcagagttggagcg<br>cccccctcacctcattattaatatagtgatgtcatcggcatgagcccaaactagtatgcctattggatgaaactgcg<br>gagggtgacgtcaagcgccgtgggacgtactaaggttggggttgggtcccgagtcggagccattACAGCCGG<br>CAAAGGTCCCGCATAGCTCAGCAGCCAAGATGGTGGGCCCTGGGAATCGGGAGATTA<br>GGGGGATTTGGGGCAGGGAGAGGACCGCGGGTGGGG
Ttyh1	ENSRNOG00000032699	>FP000238 Ttyh1_1 :+U EU:NC; range -900 to 100.<br>acacttgaacagcagcctagggatgtttgttttaggtcacgccagagactgagtgtgacctaaattcttagtgaag<br>gtctgagtgatgatttcacggtggccaggttccctctagggtctccataactgcctcccatcatctctctgtgtaactt<br>tagtagcttcccataagacccaacacaagtattttggctagaagatagctttacagtgagtggcaagagatgggg<br>gttattatatagactgaagcagtttggaggagatcgcaaacatgtgggaagtcaccatacatagatgggatgggt<br>ccctatatagctaactagggtcctattcaaagcaggtggggtccttataataaacagtcttcacaacgtgtgtaggt<br>ctttctggaagggtctgcccacacaggaggatttctctctagaaactgtgtccatatacaggttatgacaaaatgtg<br>tcgcggggaaggagacttttacctaagcagaagccgtctttacaacattaaaatgagggatttttcattggcgcgc<br>ctggctaaatgctgctcaaatactaatgacctttatggcaaggtgaaggttttacataaggataaagagcttgtgtg<br>cttcaaagagaaatcccagtatcctggctcttgtacggacagggacaaggcaggctaatccattttgctctcagca<br>agcacagcccctgcataccactgactcgcaaagaccacccgcacctgctcaggcagcaggtgggagcccaggag<br>agggcggagcaacggggcccattgctcacgccgcattgcctccggtggccaccagaggcagcactgaggcgctg<br>cctcgcctccccctccccaatctccaccccccgccctccgggctcctgctcccgggagccccgcAAACCCGGCT<br>CCGCCCGCGCCCCGCACAGCACCGAGGCTCCCGGCCCGGTCCGCCCCGCTGCCCCTCC<br>CCGGGGCCATGGGGGCACCCCCGGGCTACCGAC
Pura	ENSRNOG00000019062	>FP011240 Pura_1 :+U EU:NC; range -900 to 100.<br>ccctggtgcggctactgtcctcagcccagctccccccctctggtgagtgcgcccgcccgactgtgcggggctgcggtt<br>gggggagggggagcgggatcatctgaggccagagccgctgccgtgcgcggggagggggagcggcgggagcg<br>aggggagggacgggctaggtgtctgctgctccgcgccactgctgctggcgcctggtcctcatccccagcagcccc<br>tctgcagctaagggtttaccaccgcagcacctctcttggctcgcctgcctcttcggctctgcggtaggagacagccc<br>cgccgggtggtgggtgacactgtctgggtttgggtgtgggtcaggcccacagggaaaagggaggccgtgactcgg<br>tgtcccctctgcatcctactctgctgtgctgtgaaggcccctcccccacatcctctctttggggtcactggaaagcag<br>agacaggcctgatatctgtgctcattacgacatattccaccgcccccccatccctagtcccccgacttctcaagtcg<br>aggctgcttagggtctggccaacaggggtagcaacggcggtggcggcagaggctgcggtggcaccagaactgcg<br>acgcggcgctcccgctcccccctccagagaggggtgtagggcgagggccggcgggtgggcgcctcctggcgggc<br>aggcaggcagggccgctggcctggggaaggggcgcagtcacgtgccagcgggcgggtgggcgcgtacagtagg<br>gcgccctgctactgtactggggagtcagtgccctgttaccgggtctcgtctgtctcgtctctcccgcagatctcgcga<br>gagtggctgactggctgtgggggttgcggcggcagcaggcggagccggggagggaaagcagcggcggctGAG<br>GCGACTGAGGCGGCGGGCGGAGCGGCAGGCGGCGGCGGAGCGCAGCATCATGGCG<br>GACCGAGACAGCGGCAGCGAGCAGGGTGGTGCGGCGCTGGGCT
LOC1036 9487	ENSRNOG0000	>FP012055 LOC103694877_1 :+U EU:NC; range -900 to 100.<br>ggcttccctgagggctggcaaaatggccagtttaggaatctctcttaaggacagagcagaactagtacctctggct<br>cacaacctacgcccattcctcccccagagttctccaagcatcctcacctgcgaggaacaacagagatgcagggaa

7 (Mif)	0006589	gtgactggatggtgaccccatcctgcctctttcctcatttcacatgcacaaagcctacatgtgggggattgtaggcct tgtggacacatgtcccaggaggctcaggacacacaaaaagtctcagttgaaagtggctgggacgagggtggtact gtgtcaggggaccgagggctgactgttggtacaacaggagagcaaaagccagtaagtgatgggggcttggctaa tttcttgagcttagagaaagttcccaaggcaaggaaggattgttctccaagtacaagccatcacgtttttggctcatt gtttgaggttaaatcgtattcgctaaagtccgtcgatcctacccactggcaggagagataaggccaacctaccggt cccatcaatggcttaagtttcctctacttggtacaaatctctcagacctgaacttgctcctactaatacggttaatctg tacagcatctacttgcaatgtctcgacgaacctaatcgctagagtcaagtcctcactacctagcttattaaatgagg catcctccgtttctatcttaggaaacagagagccaatgtaatattttctagaggcacagcaagacctcggcagaaa cagcgcgctggagcgtagtcaccgccccctttgggacgtggcctgacgtcagcggaggcgtagcgaggggagga gcagcagccggcttggggcggtcctgagctgggtcacgtagctcaggtcccagacttgggtcACACCGCGCTT TACACCGTCCTCCGGCCGTCGCTCGCAGTCTCTCCGCCACCATGCCTATGTTCATCGTGA ACACCAATGTTCCCCGCGCCTCCGTGCCAG
Skp1	ENSRNOG00000005828	>FP007693 Skp1_1 :+U EU:NC; range -900 to 100. taaaggttaagattatccaacaaggtggtgcctgcctcccagcgctgagctattggaaacacagatctgaatttaa agccagcctggtctaaatagcgagttccaaacctggtttatctagtaagaccctgtttcaaacaaaacaaaacaa aacaaaaagagtataaggcgagtatatgtggtgaaaaaaaaaagccggtaatctcacgagttttagaccaacct ggtctacgcaatgaatttgcagtcactctgtgccaaacgagaccccgtcttaaaatgcaaagaaaataaaaacta agcaggtgcaccaagtcaggagtatttagagacaccttgactcaacatccgtaatttttaaagctcagttatcagt ggagtaaggttcaagtccttaattccaggtcggaaaaaaaaaaaaaaaaccaaacaaaccaaaaaccccttattt ccttgggttattaggagagtaacagtggtcaggctgaggtgactcctttaaagcacacccacccacaaccggtttt gcggtttgttctttccaggcttttcaggactggggtctttcaagcagtttaagacagcctctgcctgaaagacccttc gggtcgcgatggttccccagccggtgccggcgtccgcacggaaggagagcacggcgttgttcaggacggcggag taaagtggaagtccccctcctgctcagccacccttccacccatcgccgctcagcacctctccaggagacctctgcgg cgcccagaacctgccacccgccttccgcatgccgtcaggcactctcaacggccagcccggccggctgtgtgacgtc acgaactcggggtcctcggtgccctcttcccgctcctataaaagctgacgccgcgcggcgctgctgtAGTGGCC TTGTTCTCGAGACTTCTATTCGGTTGTGAACTCTGTTCGGCAGCCTCAGGCCTGCGGTC TTGAGACCGAGCACGGTGAGTAGCAGCCCGCGAGC
Psma6_1	ENSRNOG00000007114	>FP005130 Psma6_1 :+U EU:NC; range -900 to 100. gaagaaaaattaactcgagaaattaaaactttcttatgaagaatctcttcaaatgttttgctttaaaataagtgcttt tagagagaataccacagatctgagtatttcagtattaaatcagtaaattgttacaaatattggtgtgaacgaaactt tttttatctaggggaaaattatgcctcaacttttagattcaccaaataggagttgtgtcctggtgtgcactatgtgtag aattttagagtttgaaaaaccagttttccaggagctatttcaccgacttggaattactctccagtattcagccagcac agcaacacaggaaccgcaagttcccattacttgtatctcaacttcaacttcacttttaaggcaaaaaaacaaacca aaccaaaacaacatcaacgaaaaaccctgggccgctgtccaaagaaaagtcttgccagactagaagaaaactat tgttatttccagtggcttcttccattagccctctttcagggcttacatttaagggtggctgctgctcccccaaaacgta ggaagagaagtggcaagtgaaatgaagttaggaagaacaaagccagaactccagtaagacaagatagcaagt acaggcgccaaaactgactatcatactgtctcctgaagtcacaagtaggcaaatcacaactgcgaacttcctctttt ggacaatatgttctccagaatgcactaacggccgcgacccaaatccaccacccaaaagcacacccgaccacaga atgagctcggggccccgcccacttagaggcggtgctttggaactcctccactggaacccacccccgccagtacctt accagagcggaagcagctaggccggtgctccggaagcagtagccataacttccgggagggGCTTGTGTGCC TGGTGTGTGTGTGTGTGTGTGCGCTACGGGGTGTAGACTGTGTCTGAAATAGCGGGA ACGCCATGTCCCGTGGTTCCAGCGCCGGTTTTG
Psma6_2	ENSRNOG00000007114	>FP005129 Psma6_2 :+U EU:NC; range -900 to 100. gggtttaggtattattgttagtgacggtggtgtttttatgcttgctggtgaggatatcaaagtgtctagaaatgcata cctgtctggtcaggaacctgcagcccttcacttgcctgaactcccaagtgctaggttgtaggcatgagctgccaagc ccacttagattgaattattaaaatcacgtgaaagggctggggagatggctcagttggcagtgtttgttccccaagg acctgaggacctgaatttgaccccagtgctcacatgcagaggctgagcataggggcatatgcttaaaaagaagca ctaagggagtgaagacaggctcactggtcaccttgcctatcaggtgacctttacgacacagaacaagcctgtcgc

		aaagttcaaggtggcttctgaggaacaatgatggaggttgccctctagcctattcatgtacatgcacgcccacatg aacacggaatagatagctggaagttattatcatcagtagttaacagctgaaagatttttggtaactctggcaaaaa aaaaatattttcaactaagctaaaattaatgcaccagtctttttttttttttttttttttttttttttggtgctacttaaaaa aaaatctactccaagaagcaaatgcctgcgaagtcactggtagcaacttaatcgctaatatgttagcgaaataaat tcaagatgtcaggtcaggcaacctcatccctctcaactgagcagaacatgattagagatcaagttcggctgccatc taaatatttaaagtccattactaaaggtatctcagactgtctatgattacataagaatagaatgtctgtgtccactag gttccagtggaattccggacagttctatccgtttggcgtttctggggcagggttGAGTCCTAGGCTGAGGG GTTGGGACAAAGCATCCACCTCCACGGAACAGCTACTTTTAGGTGGATAATGGCCAAT CTCCGAAAAAGTAAGTGCCTAATTGT

Table S3

Random DNA sequences used as background for promoter analyses.

Sequence Name	Sequence
Random 1	>random sequence 1 consisting of 1000 bases.<br>gaagcctgtcactcagattgatgttacaactcagacaggctagggacttgcggtgaagaatagtctacgaacgtcga<br>aagacgttacaagaggcttgatggtttacggccacgctggacttctgttggggtgagaaacgaaggtcagccgccgt<br>ctaaagggcccgattccacctaggagcgcgatgtgataaccccgactcagcccagcggtgattgatcctacccacag<br>acaagtatctttacagtgaggaagtccacgcctggtcctacgcgcacatagttggtctatgtcacttcagtaccccgat<br>acgcacaatgcgtgacactggaggtcccaaatactcgcgacttataaaggactcgtccgtcggctaaccgcatggtt<br>atacgtgttgcggttgggccggtatcgcccgatagtgactatcggcgattcgatagaagaggagatttaccgcttgcc<br>ccggcaagcggtggcgttgcgtgttcggagcgctattaactgaaccggggacgccgggttatacacccggggtgtac<br>tatattgtgcgcgctacgtttctcacacgcgtcacaccgcttagaagcgtctactagttatcgcatgcgcccaatgatgt<br>attgcatcgagacttctcccatagcgtcctgaaggggctcgacttcgcgcatcgtttctggagggatagctgggactag<br>taccgtatcctaaggtgacctaccattggctgtcatttttaagcgctaatccctttcaggccttgtgtctcatcagactg<br>cggacccgcctaaactgatgtaggagctggccataacacccataatatagggcggccgtttcagcaaatttagaacg<br>aacaatatgctggcagtcttgtctaccggggtgtccaacataatcgaccatcatcccctgccggaattccggtggttgc<br>tgtggggttgtgttgagttttgtatgataaatatctagaatcaaagaaacaactaacccaga
Random 2	>random sequence 2 consisting of 1000 bases.<br>gtataaccgtctttagagccgggctacaccgttcgccgatatatcttttgcgggtaagaggccacaatgttaccggac<br>gcgtgtgcaggcgagctcaagcgcggcgggaagctcaaacgttacctaaagcactcaatgagttcttggttctatca<br>acgcgtccacctggccctactattgtggatagcaagcatgtcacttcggtcgtcgggtgcgtgatggtctagcaaaag<br>acacgatcgagggacgccgtgtccttctgccgcgaacgctgtcggcgagggtcctatcaagcaggccggtcgccagg<br>tgaggaatgacggtacgcggtaccagacgagaaaaaaacttatggctgaatcctctgtgatactagccatatagagt<br>ctacctcccggttcgctgtagatctacaagactggcggcaaactttgggatagcattaacaattatttgccatatgtatt<br>cttggcggcgctgactggctgtctttggaaagcacatgattgatgaaataatggagatcaaccagtgaactgtaaggt<br>tctcctcaatccaacaactccccggggtaggctatcaacgtccaggcctgaatatagcttcatcgtagaggatgtagt<br>accgaaggttaggcactttccggtctcagcgaacctcagagatgtcgttgaacattgtgcgcatcactaggcgcggca<br>cggcaatatcccgctagtgacttaatcagtaaatggaaattgggaatgcgactaatagactaagctaagcagaccca<br>gcgaccatagtttcttgcggagactgtatacgagtgcagtcagaccgtcgtacgcttagtttacacacgaccgatcca<br>ctgatgtccaggtagatgaaatggcttgctgtcactccgcccgtaattcgtagttctgcagatccacgttcccctgaatg<br>cagttcagtgcatgatcagattttagtcagcataatctatcataaacctcccaaagtggccggc
Random 3	>random sequence 3 consisting of 1000 bases.<br>acagcccaatttgtgtctacattttgtcacacatctgcgtgacgcccacttcctcgcgcatcagaccggaatcgggcgc<br>ggagaggttcattggctgcaatggtatcacatgcgtctcactggtgggtagacttctgatgctttgtagctaactctgac<br>cttcgctaaaaagtgccagggcacgactaccccatgtgtcgtcgaatagacatgcctacctcaacgtttccatcacta<br>caaagcattgcagcatggtaccactcagctcccgtatggatgcttcaacgagtacgtaaatgcgcgtcctttataaaa<br>cgtacatttgaacccaatatccaacgcctcgatcggacaagcgccatgcgtgtgagacataatcatttccggacgggc<br>gcataagcaaagtgagggtccagcggtagtccatgtggccggacgccggcacgcgtgcttaatcgcgaattgctgaa<br>aacggctacacctcacagaaagattgttgcacattggtccgctcgttctgagtttggtttcttattaatatcgaacggca<br>tgtctgtgactgttaaagttcggcaaagtcttatagaaaggattcaggtagggcgcttgcgagacttgttgagcaaga<br>gattagggatttgagaattggccattcgtcctcacggggcctgtattcactgcgtgcacgtacagtgttgcgcgctggg<br>cgatttgtgacttcggtctgtgcaattttagctggatggttaggggcggatgtgcaggtaaactaatcacagtgagtgc<br>acatggcaagcgcatatctagcagaggcccaggaaacaccgggccatgtatagaaagaatcccgggctggtttttac<br>gtccatggttgtataatgcccgtacttgcacggtgaaactcactgttctgggggcgcaacgcgggatatccaccagag<br>tttgagtgctacagattcgtcggtggcagtcgataggcggtattgaccatctgcgttcc

Random 4	>random sequence 4 consisting of 1000 bases.<br>tctccaacgaaaggacttcggcccccaatgcttgacacatggctatgattgaggtgctgttccgctttctcccgccgct<br>aatctcgtaagtagacttgatcgacgcacgtgaatccttgctccctcagctcgcaggcactccctggcggcccggggt<br>agaattcaccgcacggggcgcctcgtgctgggggcctctgaaggagccgccacgatcaagcatcgctttggtctacct<br>tccttaatggacgaacaaggagtcgcccattattccgactggtgaaaagaggtgctactgatacaggcgacattgcc<br>atacgcgtgcactggccaacaataaagccttgcgtcctgatcaatgtgaggcgcaagaggcaaccgcttattacggt<br>gtcttccgataaagatggcgtctacttgatcccatcgcattagcgggagctataaggcagtgggctgaggagccattc<br>gcagcccactgaatccgaccttagatatgtgtcccgtcgtcgggcaggcccatggcttttcagaattataagtcataag<br>gaacgttttggcactcgaacgggataaagatacatgtacagtaacttacttaattcagagtccagccccagaccgca<br>gagagctaagaatcccctacgcgccctgataatatcttttgcgtgtggtgacggtgcatagaccgtgactagacgttta<br>tccgtatctggcaggcaacctcagagcatctgccgaatgcccaaacgccccgctggtaaccttttagaccttccgggg<br>agacctgggatgggtttcgtgccgcctaccaatgatgctgaaatacgacggatacgcgacctcaaggtgagatctag<br>gacacaaacccttctactgttcttagagtagcaatccctccttagctactgtacgctgcactcccttacaaagcaccat<br>gtgcgtaggtgttagactgtttgctcctgagaatggtactaacggctacctcgagtaaaacaac
Random 5	>random sequence 5 consisting of 1000 bases.<br>tttaggctttagacgtcctccctagttgccgagcgttgcccctcgcgagctggggtagctcatttagcctcaacatagg<br>ggagtctttacactgcgacccggcgggcattccttcataacccaaacctggacggcgcaagggacccgatccgcgag<br>gagactgttgtgtctcagacgtcgagtctgttggaatcgggatcgacctcagtatcagtgatagccggtggagctggg<br>gaccccggtgctagagatcgacgaagcagaataattgcccccagagagctgtaaggtcagaagcggacgccctg<br>ggcccggatcgttggcatgatggtctgagtaggggcctctctcgtccgatcatttagagtactctcagtaccttgctata<br>atagtttcggatcatgccgaacctaggtcggatgatcgacacgggatttggttagggcaaggcgaggaaacctgaga<br>aaaactgagggattcatggattttctgttcggggatgagttcttggtttgagctggtgatccatattaagcaactgtccc<br>cacactgtatgcggactacctcatgttctcaaatttcataccacgcattaagcactggctgactgaagataagaagac<br>cacaagctcttcccccgcccgctactaacgttataaagggaaacctaaagaacctacttaggtcgctgaggcatttttc<br>tatgctatatgttgctgggatccttaaaaccaagattctggagtatacgcatggtcatacctctccgagtaggttacag<br>gctaatcccggcgttgtcacaggctacaccccggccagctaaggtgaagcgcacagctgagtcgtaaatgcagtctt<br>cctcaagttccggcatcaagcatagtgttacttgacgccacgaatataccgacgcccgctttcccattgccgtcctgtc<br>agagtaaggccctaggacttgttccagctcacatccacgttaatacgattaacgtctggca
Random 6	>random sequence 6 consisting of 1000 bases.<br>gttaaacttgttagatggcctgaactccgtacgtagagtgggatgcggaacgggtttttctcatttcatcatcgccgcc<br>aacgaagtctcggtattacctgtgacgtgttcgataggacatactcgccaccaccaggtccttctatccccctctgacc<br>ttgtccacgaggaacactatgcagctttgctaacaagtgcacacctttgacagcggacaacatcttcatagtttcaaat<br>ataaaatcggtcggccccaatatggtctcgagctgagattaatttcggatttcagttctgaatagataggtatctcaat<br>tgttgttccatacgtataccatcctgcgcgattcagaagcttatagattcacaggggcgcgtggcttatcctttaactaa<br>ggacagtttaaaccgattccagccacgcagggcgggttcgtggggtcctttcctatgcaagcaccgacggaggccca<br>tctgtgtcttgagggctgttccgaccgggtaggaatttcccgttggatgcgtgtagggcgtgcaactcaatcctcgtcg<br>gccacctattatacctcctgaaaagcatattgtgctagagtccccatagacttgctatgcactgcccagaagcatacat<br>cgtcgtggccctggtgagcttcttcgagttcgagcaccgtggatcattgcgtttaagtattcttgccaatgttatctatag<br>ggtccagaaaaatgcagcctatctgaggtcgtaggggcgagcaaaaattatacactatgaggcgttatatgcggact<br>tcgcaatacctgctatccgatagactgggtcaagccctcgcgctttccatcgcgcgtgtacggctactggcatagcaca<br>tatcagcagacgtgcacaatagcagcgtttgcccgtgcgtattcgactttgttcgaaccccaatcaggtgtttatgcta<br>gcttgcacagatgaatgatccgtcgctctcagactacccagacttgcactca
Random 7	>random sequence 7 consisting of 1000 bases.<br>gaccgcactggctagtctgctgaagggcgtgttaatttgttaccgcgatgcgacgccacgccgtcccatgctaccgag<br>ccaaaagccatgcgtatatccagtactgtgccaagaacccgggttcagccgtctgggccacagggtctacgcctaag<br>taaatgtgaaagtcaagcctcgtcccaatctacgaaactgtctgaatcagttgcatcctggcagaaacacttcccagt<br>gcatataactattcgtacccttagtttcacctgagcttataagcctggttcagttataccaacgttggatagacaagtta<br>gcgtcatgcgccttttacgttagcagactgactactcccccgtcccacattgcaagacgcacggacacgtatgcctggt

	ccctattacgtgtgtgaaccagttgattagtcgaaaacttaagctgcagggctgcatttttagcgagcgctgtcttcaa<br>agcggggcccctatggggctgaccacagccggcccaggagcacaagtcaactgttggctctggcattgataagaag<br>gcgccgctaccctgcaaaatactggaacaacctctacgttagttggtaatcaccgcagtccgcccgggatacgatga<br>gcgtcttcggcgcagggtataattattgccgtcacgcccgagctgatgggtatggactcgctgacgcgcgcgctgcat<br>ttgtactgcctagatgaatttggaggcgtaatcatgcgccgcgttctacacaactcagtgtacatgtggaatccatctg<br>cggcctatcccttattaggctccggaagaacgtcggtcggtcatgaggtgagaatatcccggtttggaacttatctaaa<br>tcgggagatcccgaaagcatggagcgcggggcagtggagagcatactgccctctaaataatccctctgtgttcccgtt<br>ccaagaacgtttgagtggagtaatgccccccttgctaaatttcggaagcggtaaacgcaagg
Random 8	>random sequence 8 consisting of 1000 bases.<br>cctgcctcgggccggatccgtttcatcagaaatcggacacatttctggcagcccaccctaatagacggtgttggcatg<br>agtctgttgacattgcggaccgcgtcctcgggcgacgtacttcgggagccgctgtgtgcgcttgacatttgcgaggtat<br>acgcaatcacatactggatacttcttccatatacgaagctctactggatacgtggggagcaactagctcataggattc<br>cagttaccgtggatccctgggaacatttatatcggataatacctgtgacgcggtatcctcggaaagcaccctcgtacg<br>cacgtgatcccgccttgcagttgcggtatacagcgggcacttttataggctagggtgtcatagtcttcccaagataaca<br>cgcagtattgggttcggaagccctataggaatctatttagtcgaaggacataacctctggctaatcgcacataagagg<br>tagtatacattcacggaacgattactcgagccgtactgagctattgtcgctgagcatccgtcttatcgcaccgagtctc<br>acatacatgcgtaagagctctcgaaagcatgtataacctcattagtgatactgtcctccttttgcgacattcttgtcgta<br>gcaagctagaataattttgcgttcgtatacgtagctaggaacaggggaccggatagtataacgctaccgtcaggaaa<br>cctgccagcggcgagtgtgaaatgccaatgatgcgcctagtgcctccaggtctggtcgctcgccgcccgacagttatt<br>atagtgctccatggggcaatccacatccttctgatgttgccacaacatctaacaccgacttaagcccgtaattatgccc<br>acgtaaacctgggcgaggcaagccgatgaccttgaccagactcgaaacgctgcattagacgctttatcatctcttaag<br>agttaccgccacatctgtataggcagttaccccgagttttcggtattaatgacattgca
Random 9	>random sequence 9 consisting of 1000 bases.<br>ggattttatccacgttctgcaatgacacccccagcttatggtaataagcgctcagtaccaagctgaaggcacgcctgc<br>ggcggggttgtatttagttgcaaatcgtttcagactcaggcaggttttaatgcgacgccgcaatctgaataatgcttat<br>acccccatcagaggttggcaatgcaatctaggcctacacgttataggtaaagtaccaaaggtggtttgtctgccagat<br>cggtccccatattgaaatcgggggctaggcccgaagtacgggagcccacctaaatgcatcctcaacgttggtctctcc<br>ctatgttcaattcggtgttgcgttcagtttcttctaatccttagcggcagggggcatcagcacccaaactgagtcgaggt<br>cattcttcagaagtaccacctatgggacgggctagcgcgtcagatcagtgtaagcgccggaccacctggcacgctgc<br>tgtaaggtcgcagcagtaatgcacatgaaatcagatcggacatccacattatggcgtggagcgcgtgtggacaataa<br>aaatctgttaccgggcttgaatagctgcgtgctagaaccgcccgacgtcaagattacaatttggtggctcgggtgatc<br>aacacttctggtcacgatcactacacgtcaacgttttgtgtagcccgtgtgttgctaagcttttgtaggttattttaagtg<br>tggccgtctcacggtccaccgttacttacgcccaaattagcaagcccctgaaccagcgtctacggcaatgtaaggatt<br>aatctcagtttcggctttcttaaagaggattctagttgccccggaattcgtgatcaatggggtgcatttgaaaagcgta<br>cctttcgcgccccgcaagtttatactaaacctcatccaatagcttcatccgtaacggaagaggtactctctagggtggc<br>acgtacgaatgcaaacgccagccagttgtgggggcggtatcgcgtgacggaactcga
Random 10	>random sequence 10 consisting of 1000 bases.<br>caatacgatacttcacaacacgtttaaagttccacgtaactaacggcgcggtactttggcaggttacgtttgcgtaccc<br>acgtatgtcgacctagaccctcccggacccgatttacggctcaatggttcgtggagcaaagttcaactcaagatttact<br>attccttgttgaagcttatccatgatcgtaacagtagcatcctgtaaccgttaagatcaattcttggtaggaaagcattc<br>agcctagccgcagggtagtcgtgacttagttgtttagcttccagaataccattaccgcatcatgaccgcgttgcgttag<br>atttgtacgcttcaaattaataccggaaaatttagctccacaaaggctgtcgagcgtcacacgggagaccacgaaca<br>gacctgagatacgctactagtagcctcgatcgattacgataagttctaatatataagcggacctcatctcgtgccttcc<br>ttccgcacgggcagacacaccaagaaaacccgctaagaatcggacgagtgcatgggggcgaggttttagccctcca<br>cgaaaccacggataatgggtacatacagcatgatgacgggaatacctgggcggagtataggaatgagccctcaatg<br>actcgaagggttgataatgaggatgtggaaatgacggtcaatgctgcctggttcgctttagcgccgggtccttatgtgc<br>gaagctacaccgtcgtacggcataccattgagcaggacgcatccccgtggtgagattgctacaagagtcacgctaag<br>cgccgttcattgttgttaagtaacagtaatggacacggccctaacacatctatttatatccaattctgcgcggggtagc

	ttctcgactaaaatctacgagcggggacaccgtggctacgctatccgtctgtctgtatgtgaacgcctcatatagccta<br>gggacaatatcccgcggagaaccaaaagcctgaaccctcgctccaagtattacttgggcca
Random 11	>random sequence 11 consisting of 1000 bases.<br>cattcgtagcggagcctagcgctgggcgagatttggcggggacgggtccctaagcgaatgacaggtcttaaagattc<br>agtgtataacgaacttttattgttcgtgtcctacggactgttgtatgtgcgacatatgccctgttgcttcatggagatga<br>gatgtaatgtcgcggggaatccgcaaattattggggaagcgcatgtcctgacacgcagcgtccctctgatctcactca<br>acatatcacaggtgataacttaaggactgatcggatggaagtggtataatcgaccccagacccgcgatcttagcccc<br>atatagtagagcgtcttgtgtctgttactgaaccttctgatactaatcgtaatctgggaatcggaacctatatcccatgg<br>gagacacggaggaactgttggagttttagtatcacatgtatttagaatggacgctggtatcagatacagagctccgaa<br>agtggttccacgccaatttcacgtacgtgagacgatcataactatcatacgttacccacgtacacctgaggtagaacc<br>atacggggtagccactcgccagtatcttcgtccctgcagatcaattcgcccgatacccgacttcaggattttctcagaa<br>cttatgacacactgcgtgtacagttcgcggtgttatagacccagcgcgcccccaagtcagctattcaataggcattgta<br>cctacacgatgattctcgccctgcgcttccagggccttggagtaaacggggcaatttaaaaggcacctatagtagtgc<br>ggtaacacgcgatgaaacgccaggaaagcgcgaatcacgagctccggagcggcggctttgggcgcgcacgtaaca<br>cgtctatctttattgatctcaaagcccacggacttaggctacagactcctctcacattagaataacatttggtggggt<br>aactagcatttgtggccggttcgagagagggcgacgttcccgtagcactttatttcgggatg
Random 12	>random sequence 12 consisting of 1000 bases.<br>agcgttctttgttcaagaaagctccctcgtcgagtcgaagacactcgggccgcttttaatgaagccgttccaacaatta<br>ctttatcgcaattgccgttataggatacctcatcgaagatattacatagggcatcttgcaacctcgtttattaacgccat<br>actcgttttagaaggggtccgggatcacctggcaatttggagcaacgtagtagacaaaaccttgacatgtaatccacc<br>ctgcgcaccgttagttttccttggagcgctcggctaacattatacaacgccattcaccttatatgaactacccgtataga<br>caacgattgttgagcgttagtgtcggacccttcagatttccgctttggtgctacagagcaagcaggcaaggaacgagg<br>ccgagcttgtgatgtaaccccccatcacatactaaatcggcggcacctagctagccattttgcccataatccaaattgtg<br>ttcgctccagtacctgatagtaatcaaaacccctctatgccgaagcatacaaaggccctagctaaatgtgcctactac<br>aatgactcttttatgattctatttagatccgcaagcccttgcggtttggggctaccgcggctaacttaacgacaatcatt<br>cccccactcgcggtacgaagtagaatttgtagataactgtctgccttgaatgaacccgcgagacgtcaccaggtccgc<br>ggggacgcagagtcaacgggaaggaattagcccgttaattctgcgtgcctctcgtaaccaaccaagcgcccgtggac<br>cgcgatcgggttgacgactgccctgcacttaggtagtcgacgtgactaactttataagtcgctccctggtaggctcgga<br>gagctcatgctagagcgcaatggtaaatgattaagcaccaatgcgagacttcgcaccgcaacttgtccgtctttaaat<br>ggcatgagttctcatgcgttcccaggactttaccaaacgcaagatctttgccgagg
Random 13	>random sequence 13 consisting of 1000 bases.<br>agggtgtcttgatggtggatctggtgctgggaatgcagtgctgtaactacctactggggatgtcccccatgtacaatca<br>agttcatgtgaggagagcaacacggtgagtattccgaaccacgacttaagactaatagtaaaagaattaggttatcg<br>ttgaacaggtcggccgcgatactgtctcatatctggttcgatagcgcattatcgagcaatagaacgcgtcagagagtt<br>cacaactgttgttcaaaggtcacactttataaagtacctatgagttacgcaagtatgcaaagtagtaatgtttatcact<br>ttaatttacacctgaggtccagtatttaaaatgtatgagttttgaacccatgccacgggtcttaaatcctactagttcag<br>agattatcggacagttactacggctggggatgccgtcgaatcctctcggatacgctcgtttattatggctgatatcattg<br>ccccggttagcactgcatcgggggcactgatgtgtggctcctcctcggtccgttccgctgcttgcaatggtaactggctt<br>ttgtgttttcgtgcaatcaattggtgtcgttctatacggtttgcgatgttcgagacgttcgcgatgcggtgagagcgatt<br>acgtgcaaatttcagggcgacagggaagttagatgccatgaacggggttttccgttattagtcctctgggtaggggtt<br>cgcgacctcgtctcaagacaacggggtacaacttcatcccgcgatcccaacgaaattactgcgtattgcaatccggat<br>ggggcctgagaaaaacaccttcggcctcctctatagcttaacctcctgtcttatcttaaactcaacgcacttcaccgac<br>gtattctgcgccggggtaaacgcccaatccttgctaaaactatgctcaccccacaccgattctcataagcaactccttc<br>tcttggactggacattgccgcggcacactttgggatcggcgccagggacggtt
Random 14	>random sequence 14 consisting of 1000 bases.<br>agttaaagccatgagacggtgaaaattgggtagtactcctataaataaggttaacttctgtagctgccatcgaggtctt<br>aatcaggaatgagtaattccgtgtgagggatacacagattccgactgaacatttttgtagggccggtaccggactaca<br>tttcgcggctgactcaaacgaatggatcaccacaggtcaattagatccccaaccgcttactcctcacacgttatatcta

	aatcgcgccataaagcaattcctgttgaggatccagtcgcatcttggtctatacgtaaaccacatgctaaagcccacg<br>ggagatccctatacctggttatcattccctaggagactctaggctaatcgtgatatgtcgccttttaaagggtaggtga<br>taacacccctctctgtgtggtagcctggcaccctcaacgatgagctgcacgcgctccaggcggcataacagaatagg<br>atcttgacgctttctagattctatagcccactatcgtctaacatacttataccggattgctgaaatcgttacggcctaatt<br>gaccccgggcaactacgctgggtcaggtgttagtggttccgagaaaccaccgtggtggttgtagttattgactagacg<br>aatggccccgatagtcgtctgtaggcaggtcacactggagatcgtatttcacgaggttaatagtgaattcagatatag<br>atcgaaatgtactgctgcgtacatccgtgacgtaggctcgcctatctgaacaaggcagacgcctatttggacttcgcc<br>cagatcaacttcgaatcattctcaggtggtggggatgattttatcgtttaggcagcgtccggacgtcaacgaatcacgt<br>ggaagttgttacgaacaagtacgccagctcgcttactggctgaccggccgctttgacatacgtagtatggaaaatgta<br>atctttacagcctttgcctgaggaagatatgggtgcaggctaccgtcctgtcactctt
Random 15	>random sequence 15 consisting of 1000 bases.<br>ctccgcggtgagtcggtcgctcgcagtgcgggccacctgctagatgaggtcgtccattggttcgcgggtcacgacccg<br>cacataaactgcgcaagagtttcgttccctggcggtcacgcgcttttcaaagggccatcatctcaacatcgtcggactt<br>agaagatgtgctcggtaccggcacttaaggaacaacatgttaattacactttccaataacggacttgtagtagtgcca<br>agaagtaatggttgatccggtcacggagtgaaccagtctcccgcacgtattcttcctcgatcagagcgaaaaaatacc<br>ggaatagatgaggattagtgctccgccaagatagagcgaaggccgcgccacatggggacagatttctctatatgcta<br>gttgttggttgcggagccgcgggggttagacaaccatgccgagcctaggtcccctagcgcccgcttcccctgtcttcag<br>cgtattccgccttgtgatggccaccagctccctattgtactctcgctgtttcctagcgggtggatgatggtgaaattcgt<br>ctactattgcgtagtgtacgatggaattgaccttctatttttctgaaacgcggcgggtaatatcgcaacgagttaaaac<br>gtatatcaaataatcctgcagccgcaaggaacgtttttgcatggtacggcgaataccctctctttgacttgtgaaagtg<br>cgcatacgtttggttgattaatcctcaagagtgtgatttcgttctgaataatctgaggcaaccccccttcatagcttacat<br>caccaacagtcatcatcggaacgccgcgacagacgacatctagtcatagtttttcgtagataggaacctcgtttcaat<br>tgtcgctctcggaactggctttttcacggcatgatcagaagctatcttgcggccctaagtagggtaccccaattatgag<br>agcgcaacgaacttctggactgtatccatcgaaacttcgcgcagtgcactctatat
Random 16	>random sequence 16 consisting of 1000 bases.<br>cgagagtactgggtacttaagattcgtcacatctacgcctcgactcatttgtctggtcttaacgtcgtatacctctcttcg<br>caaactcgcaacccggcgaggcacccgcccttaggccctatagttgagagcccagttaacgagtaggtagccgtttt<br>agtcaggaccagcaggatagagtgcgccctcttaccaaactgccagtatatacgtctgtggtggaacgtacacgttcc<br>catctggtcttactagggacaatcacttataggccactacctgggcaagcacgaccgaggtaacaaactgcttttaac<br>ttctattgagcagaccaaagcgtgagaaagctcgaaaggccattgtcagtttggttacaacataatacttctatgtgtg<br>tgggaaggtcatttcacgcgtataagtctgccctgtgagggcgtgccatgcaacctggcgaccaccgcccttttggtgc<br>tctgtccatctctccatctgggttcacaattagtctccagcctcaattaagcgttagctgtcagccatagaacaggggc<br>gaagtagcggaccggcgctcattgtcgcgattcacgaagctgcgagatggactgattgaagaagcagctgatcttcg<br>agacaaatgcttctatggtgcccgacacttggtcaagaaaggagctgggtgtattcatataaagtttactagttgagc<br>ctgacgtcgccggagcatcttcatcacgataaagacacagccattcgctttaccctccctactaagacgaaaaaggc<br>gcctgggggagggggatacatcgacgtggttcgcatgtacgtacatagtaccaagcttgaggtgaacgctaatagtc<br>ttctgaatggaaagacaatccatagaatgtcggctattcaacacttcgtatttggtttttgcgacgtcagtaagacaaa<br>cccagtcagtttcactattgcattttatgggaacgtcaggctgtatatcacggtgggatta

Table S4

TFBS Enrichment results

matrix identifier	ratio	P-val
V$GC_01	1466667.75	2.42E-06
V$MAZR_01	1466667.75	2.42E-06
V$LBP1_Q6	1400001.00	4.35E-06
V$IRF1_01	1133334.37	4.57E-05
V$SP1_Q6	1000001.00	1.48E-04
V$ZIC2_01	933334.31	2.66E-04
V$PAX4_03	933334.31	2.66E-04
V$HFH4_01	866667.68	4.80E-04
V$MAZ_Q6	866667.68	4.80E-04
V$MYOGENIN_Q6	800001.00	8.64E-04
V$CREB_Q4	733334.37	1.55E-03
V$SP1_Q2_01	733334.37	1.55E-03
V$ELF1_Q6	666667.68	2.80E-03
V$PAX_Q6	666667.68	2.80E-03
P$Alfin1_Q2	666667.68	2.80E-03
V$COUP_DR1_Q6	666667.68	2.80E-03
V$GLI_Q2	666667.68	2.80E-03
V$E12_Q6	666667.68	2.80E-03
P$ABI4_01	600001.00	5.04E-03
V$HNF3B_01	600001.00	5.04E-03
I$BRCZ4_01	600001.00	5.04E-03
V$E2_Q6	600001.00	5.04E-03
V$TST1_01	600001.00	5.04E-03
F$FACBCA_Q2	600001.00	5.04E-03
V$HEB_Q6	600001.00	5.04E-03
V$SP1_Q4_01	600001.00	5.04E-03
V$E47_01	600001.00	5.04E-03
V$AP4_Q6_01	533334.37	9.07E-03
V$FOX_Q2	533334.37	9.07E-03
V$SPZ1_01	533334.37	9.07E-03
V$MINI20_B	533334.37	9.07E-03
V$CETS1P54_01	533334.37	9.07E-03
V$NFY_Q6	533334.37	9.07E-03
V$NGFIC_01	533334.37	9.07E-03
V$VJUN_01	533334.37	9.07E-03
I$ADF1_Q6	13.59	3.91E-04
I$HB_01	13.59	3.91E-04
V$HNF3ALPHA_Q6	13.59	3.91E-04

V$AP2_Q3	12.79	6.67E-04
V$MZF1_02	12.39	1.37E-06
V$FOXO4_01	11.19	6.66E-06
V$SP1_01	10.39	3.25E-03
V$EGR_Q6	10.39	3.25E-03
V$KROX_Q6	9.59	5.47E-03
V$MUSCLE_INI_B	9.59	5.47E-03
V$TFIII_Q6	9.11	3.52E-10
V$AR_Q2	8.79	9.16E-03
F$DDE1_B	8.79	9.16E-03
V$FOXO3_01	8.79	9.16E-03
V$NRF2_Q4	8.79	9.16E-03
V$NFAT_Q6	8.79	9.16E-03
F$STE11_02	8.79	9.16E-03
V$FOXD3_01	7.46	3.20E-05
V$HFH3_01	7.19	1.09E-03
V$FOXO1_01	7.19	5.23E-05
V$SRY_01	7.19	5.23E-05
N$DAF16_01	6.66	1.38E-04
V$ETF_Q6	6.49	6.13E-10
V$NFAT_Q4_01	6.39	2.88E-03
V$ETS_Q4	6.39	2.88E-03
V$ZIC3_01	6.39	2.88E-03
V$VDR_Q3	6.39	2.88E-03
V$PPAR_DR1_Q2	6.39	2.88E-03
V$HFH8_01	6.39	1.30E-07
I$TTK69_01	6.19	2.93E-03
V$CEBPGAMMA_Q6	5.99	4.64E-03
N$TRA1_01	5.19	7.81E-09
V$CIZ_01	5.06	5.99E-10
V$FOXO1_02	4.99	4.69E-04
V$HNF3_Q6	4.79	1.53E-04
V$DR1_Q3	4.79	3.24E-05
V$IRF7_01	4.39	1.77E-03
V$AP2ALPHA_02	4.31	5.66E-04
V$GCM_Q2	4.26	8.77E-03
V$MEF2_02	4.19	2.72E-03
V$RREB1_01	4.19	2.72E-03
V$FOXJ2_01	4.17	1.91E-08
V$ETS_Q6	4.15	8.68E-04
V$ETS2_B	3.99	4.17E-03
V$PU1_Q6	3.86	6.47E-04
V$LDSPOLYA_B	3.81	1.32E-08

V$AP2ALPHA_03	3.79	6.34E-03
F$RAP1_C	3.59	9.56E-03
I$GAGAFACTOR_Q6	3.59	9.56E-03
P$PCF2_01	3.59	9.56E-03
P$TGA1B_Q2	3.59	9.56E-03
V$AP2_Q6	3.52	1.42E-07
V$FOXO4_02	3.51	4.53E-03
V$AP4_Q5	3.35	1.25E-04
V$MAF_Q6_01	3.19	9.95E-03
V$LYF1_01	3.19	9.95E-03
V$MEF2_Q6_01	3.19	9.95E-03
P$RAV1_02	3.15	3.79E-07
V$FAC1_01	3.09	1.62E-03
V$MINI19_B	2.99	2.34E-03
P$MADSB_Q2	2.95	8.12E-04
V$ETS1_B	2.85	6.96E-03
V$SP3_Q3	2.85	3.38E-06
V$HNF4_Q6_01	2.79	2.04E-04
V$AP2ALPHA_01	2.76	4.09E-04
V$HELIOSA_02	2.73	8.20E-04
V$DR3_Q4	2.66	4.77E-03
I$BRCZ3_01	2.61	2.31E-03
V$MZF1_01	2.59	1.61E-03
V$NRF2_01	2.59	1.61E-03
F$STRE_B	2.58	2.00E-04
V$AP2GAMMA_01	2.55	1.65E-07
F$STRE_01	2.53	1.96E-04
F$GCR1_01	2.44	5.30E-04
V$GABP_B	2.39	5.11E-04
V$CETS168_Q6	2.32	2.35E-04
V$HAND1E47_01	2.30	1.35E-03
V$TBP_Q6	2.29	5.61E-08
V$FREAC7_01	2.26	8.07E-03
V$TATA_C	2.26	1.28E-03
P$AGL1_02	2.21	7.61E-03
V$HNF4_01	2.17	7.15E-03
V$IK1_01	2.10	2.90E-03
V$E2F_Q2	2.07	5.84E-03
V$COUPTF_Q6	2.05	8.30E-03
P$PBF_01	2.04	3.58E-03
I$EVE_Q6	2.03	2.37E-03
I$DL_02	2.02	7.71E-03
V$CP2_02	1.98	8.49E-04

V$HNF1_Q6_01	1.92	5.91E-04
I$E74A_01	1.89	1.05E-03
V$CREL_01	1.89	1.70E-03
V$GR_Q6	1.84	3.02E-03
V$HMGIY_Q3	1.77	1.73E-04
V$ELK1_02	1.72	2.86E-04
I$ANTP_Q6	1.70	1.47E-03
P$DOF3_01	1.63	3.13E-03
V$PEA3_Q6	1.63	3.72E-03
V$MTATA_B	1.60	8.62E-03
V$P300_01	1.60	9.41E-03
F$ADR1_01	1.58	5.24E-03
V$BARBIE_01	1.53	7.15E-03
V$SMAD4_Q6	1.52	7.88E-03
V$ZIC1_01	1.50	8.01E-03

Table S5. Acquisition parameters for the targeted detection of proteins potentially altered by H_2O_2 treatment and reverted to control condition by MOE. Protein entry number, peptide sequence, expected chromatographic retention time (min), normalized collision energy (NCE), *m/z* value of the precursor ion and *m/z* values of 7 precursor-to-product ion transitions of target peptides are listed in the table.

					Product ions (*m/z*)						
**Protein entry (UniProtKb)**	**Peptide sequence**	**RT (min)**	**NCE**	**Precursor (*m/z*)**	**Product 1**	**Product 2**	**Product 3**	**Product 4**	**Product 5**	**Product 6**	**Product 7**
sp|P46462|TERA_RAT	NAPAIIFIDELDAIAPK	23.4	25	604.336	971.5	614.4	315.2	244.2	186.1	467.3	580.3
sp|P46462|TERA_RAT	LEILQIHTK	13.8	16	365.557	739.4	626.4	498.3	385.2	248.2	243.1	356.2
sp|P37377|SYUA_RAT	EGVVHGVTTVAEK	9.5	19	442.574	804.4	648.4	547.3	446.3	347.2	276.2	187.1
sp|P37377|SYUA_RAT	TVEGAGNIAAATGFVK	15.3	26	753.402	1305.7	1176.6	1048.6	764.4	693.4	622.4	201.1
sp|P62138|PP1A_RAT	EIFLSQPILLELEAPLK	23.2	32	977.069	1235.8	799.5	670.4	428.3	357.2	243.1	390.2
sp|P62138|PP1A_RAT	AHQVVEDGYEFFAK	15.0	23	547.263	641.3	512.3	365.2	209.1	337.2	436.2	535.3
sp|P35565|CALX_RAT	GSLSGWILSK	17.2	19	524.295	903.5	790.4	703.4	646.4	588.3	701.4	814.4
sp|P35565|CALX_RAT	TSELNLDQFHDK	12.9	21	482.900	902.4	789.4	674.3	546.3	399.2	262.1	189.1
sp|Q63941|RAB3B_RAT	LQIWDTAGQER	14.4	23	658.833	1075.5	962.4	776.4	661.3	489.2	242.1	355.2
sp|Q63941|RAB3B_RAT	TITTAYYR	10.7	18	494.759	774.4	673.3	572.3	501.2	338.2	316.2	488.3
sp|P55161|NCKP1_RAT	AINQIAAALFTIHK	19.5	21	504.296	971.6	900.5	829.5	758.5	645.4	284.2	185.1
sp|P04692|TPM1_RAT	IQLVEEELDR	14.6	22	622.330	1002.5	889.4	790.4	661.3	532.3	242.1	355.2
sp|P04692|TPM1_RAT	SLEAQAEK	6.7	16	438.227	675.3	546.3	475.3	347.2	276.2	201.1	330.2
sp|P17764|THIL_RAT	DGLTDVYNK	11.5	18	512.751	739.4	638.3	523.3	424.2	261.2	173.1	286.1
sp|P17764|THIL_RAT	FANEITPITISVK	16.9	24	716.906	1100.7	971.6	858.5	757.5	219.1	333.2	462.2
tr|B2GV99|B2GV99_RAT	EAFQLFDR	16.5	18	513.256	825.4	678.4	550.3	437.2	290.1	201.1	476.2
tr|B2GV99|B2GV99_RAT	HVLVTLGEK	10.8	18	498.298	858.5	759.5	646.4	547.3	333.2	237.1	849.5
sp|P0C5X8|TTYH1_RAT	TELTTLEEVLSER	20.2	26	760.396	1289.7	1176.6	974.5	861.4	732.4	391.2	231.1

sp|P0C5X8|TTYH1_RAT	ALASIHSQLQGLER	13.5	22	508.283	930.5	843.5	715.4	602.3	474.3	304.2	185.1
sp|P86252|PURA_RAT	GPGLGSTQGQTIALPAQGLIEFR	20.0	32	771.085	1030.6	933.5	862.5	734.4	322.2	155.1	212.1
sp|P86252|PURA_RAT	FFFDVGSNK	17.0	19	530.759	913.4	766.4	619.3	504.3	405.2	295.1	914.4
sp|B2RYG6|OTUB1_RAT	LLTSGYLQR	13.0	19	525.801	937.5	723.4	579.3	416.3	303.2	227.2	328.2
sp|B2RYG6|OTUB1_RAT	FFEHFIEGGR	15.0	18	413.537	678.4	531.3	418.2	289.2	295.1	561.2	708.3
sp|P04906|GSTP1_RAT	EEVVTIDVWLQGSLK	20.5	29	858.464	645.4	532.3	358.2	457.2	558.3	1071.5	1184.6
sp|P04906|GSTP1_RAT	FEDGDLTLYQSNAILR	17.8	31	927.965	1178.7	1077.6	964.5	801.5	673.4	288.2	277.1
sp|P11661|NU5M_RAT	STSITQTQLSK	9.4	21	597.322	1005.6	918.5	805.4	704.4	576.3	234.1	189.1
sp|F1LQ48|HNRPL_RAT	TPASPVVHIR	10.0	16	359.545	720.5	524.3	425.3	199.1	270.1	357.2	553.3
sp|F1LQ48|HNRPL_RAT	QPPLLGDHPAEYGEGR	14.6	24	579.285	781.3	181.1	478.3	752.9	781.4	747.4	243.1
sp|P30904|MIF_RAT	IGGAQNR	3.7	14	358.196	602.3	545.3	488.3	417.2	289.2	171.1	228.1
sp|P30904|MIF_RAT	LHISPDR	8.0	16	419.233	724.4	587.3	474.2	251.2	364.2	451.3	663.3
sp|Q6PEC4|SKP1_RAT	TDDIPVWDQEFLK	16.9	23	535.931	664.4	536.3	407.3	260.2	445.2	542.2	641.3
sp|Q6PEC4|SKP1_RAT	ENQWCEEK	8.4	20	561.730	879.4	751.3	565.2	405.2	276.2	244.1	372.2
sp|P40307|PSB2_RAT	NGYELSPTAAANFTR	19.5	23	537.930	577.3	664.3	761.3	595.3	594.3	705.3	705.3
sp|P40307|PSB2_RAT	FILNLPTFSVR	18.3	23	653.880	706.4	361.2	175.1	410.2	374.2	601.4	670.4
